# How Noisy Adaptation of Neurons Shapes Interspike Interval Histograms and Correlations

**DOI:** 10.1371/journal.pcbi.1001026

**Published:** 2010-12-16

**Authors:** Tilo Schwalger, Karin Fisch, Jan Benda, Benjamin Lindner

**Affiliations:** 1 Max-Planck Institute for the Physics of Complex Systems, Dresden, Germany; 2 Division of Neurobiology, Department Biology II, Ludwig-Maximilians-Universität München, Munich, Germany; Gatsby Computational Neuroscience Unit, University College London, United Kingdom

## Abstract

Channel noise is the dominant intrinsic noise source of neurons causing variability in the timing of action potentials and interspike intervals (ISI). Slow adaptation currents are observed in many cells and strongly shape response properties of neurons. These currents are mediated by finite populations of ionic channels and may thus carry a substantial noise component. Here we study the effect of such adaptation noise on the ISI statistics of an integrate-and-fire model neuron by means of analytical techniques and extensive numerical simulations. We contrast this stochastic adaptation with the commonly studied case of a fast fluctuating current noise and a deterministic adaptation current (corresponding to an infinite population of adaptation channels). We derive analytical approximations for the ISI density and ISI serial correlation coefficient for both cases. For fast fluctuations and deterministic adaptation, the ISI density is well approximated by an inverse Gaussian (IG) and the ISI correlations are negative. In marked contrast, for stochastic adaptation, the density is more peaked and has a heavier tail than an IG density and the serial correlations are positive. A numerical study of the mixed case where both fast fluctuations and adaptation channel noise are present reveals a smooth transition between the analytically tractable limiting cases. Our conclusions are furthermore supported by numerical simulations of a biophysically more realistic Hodgkin-Huxley type model. Our results could be used to infer the dominant source of noise in neurons from their ISI statistics.

## Author Summary

Neurons of sensory systems encode signals from the environment by sequences of electrical pulses — so-called spikes. This coding of information is fundamentally limited by the presence of intrinsic neural noise. One major noise source is “channel noise” that is generated by the random activity of various types of ion channels in the cell membrane. Slow adaptation currents can also be a source of channel noise. Adaptation currents profoundly shape the signal transmission properties of a neuron by emphasizing fast changes in the stimulus but adapting the spiking frequency to slow stimulus components. Here, we mathematically analyze the effects of both slow adaptation channel noise and fast channel noise on the statistics of spike times in adapting neuron models. Surprisingly, the two noise sources result in qualitatively different distributions and correlations of time intervals between spikes. Our findings add a novel aspect to the function of adaptation currents and can also be used to experimentally distinguish adaptation noise and fast channel noise on the basis of spike sequences.

## Introduction

The firing of action potentials of a neuron *in vivo* is a genuine stochastic process due to the presence of several sources of noise [1]. The spontaneous neural activity (e.g. the firing of a sensory cell in absence of sensory stimuli) [2], [3] as well as the response of neurons to stimuli cannot be understood without taking into account these fluctuations [4]. Moreover, noise can have a positive influence on neural function, e.g. by stochastic resonance [5], [6], gain modulation [7], decorrelation of spiking activity [8], enhancement of signal detection [9], or fast transmission of noise coded signals [10], [11]. For these reasons, reduced stochastic models of neural activity have been suggested [12]–[14] and analytical methods have been developed to calculate the statistics of spontaneous activity and the response to periodic stimuli [15]–[17]. Studying such reduced models allows to relate specific mechanisms with certain statistics of neural firing. Vice versa, analytical expressions for the firing statistics of model neurons may be used to infer unknown physiological details from experimental data.

Spike-frequency adaptation is another common feature of neural dynamics that, however, is still poorly understood in the context of stochastic spike generation. Associated adaptation currents which act on time scales ranging from 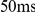 to seconds are ubiquitous throughout the nervous system [18]. Prominent examples of adaptation mechanisms include M-type currents, calcium-gated potassium currents (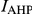), and slow inactivation of sodium currents. Functional roles of spike-frequency adaptation include forward masking [19], high-pass filtering [20]–[22], and response selectivity [23]–[25]. If the neuron is driven by fast fluctuations, adaptation reveals itself in the interspike interval statistics of neural firing, most prominently in the occurrence of negative correlations among interspike intervals [26]–[31]. These features can be phenomenologically captured in generalized integrate-and-fire (IF) models via introduction of a slow inhibitory feedback variable, either acting as a dynamic threshold or as an inhibitory conductance or current [28], [29], [32]–[34] or in even more simplified models [35]–[37].

In previous studies on stochastic models with adaptation, fluctuations were considered to be fast, e.g. Poissonian synaptic spike trains passing through fast synapses or a white Gaussian input current representing a mixture of intrinsic fluctuations and background synaptic input. In particular, the dominating intrinsic source of fluctuations is ion channel noise [1], [38]–[40]. This kind of noise is not only contributed by the fast ionic conductances, which establish the spike generating mechanism, but also by the channels that mediate adaptation currents. If the number of adaptation channels is not too large, the stochastic opening and closing of single channels will contribute a fluctuating component to the adaptation current. This noise contribution, which was so far ignored in the literature, and its impact on the ISI statistics is the subject of our study. Here, we only consider the simplest adaptation channel model which corresponds to an M-type adaptation current. Our results, however, also apply to other sources of noise emerging from a slow adaptation mechanisms as, for instance, slow 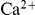 fluctuations in the case of calcium-gated potassium currents (see discussion).

In this paper, we analyze a perfect integrate-and-fire (PIF) model in which a population of 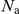 channels mediate a stochastic adaptation current. We approximate this model by simplified stochastic differential equations (diffusion approximation). For slow adaptation, we are able to show that the latter is equivalent to a PIF neuron driven by a slow external noise. As a surprising consequence, pure adaptation channel noise induces positive ISI correlations in marked contrast to negative ISI correlations evoked by the commonly studied combination of fast noise and deterministic adaptation [28]. Furthermore, the ISI histogram is more peaked and displays a heavier tail than expected for a PIF model with fast current noise. Our results for the PIF (positive ISI correlations, peaked histograms) are qualitatively confirmed by extensive simulations of a conductance-based model with 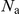 stochastic adaptation channels supporting the generality of our findings.

## Results

Our main concern in this paper was the effect of noise associated with the slow dynamics of adaptation on the interspike interval statistics. Specifically, the noise was regarded as the result of the stochastic activity of a finite number of slow “adaptation channels”, e.g. M-type channels ([41], [42]). We contrasted this slow adaptation channel-noise with the opposite and commonly considered case of a deterministic adaptation mechanism together with fast current fluctuations [28], [29]. In both cases, the spike frequency and the variability of the single ISI were similar, although the sources of spiking variability were governed by completely different mechanisms. In a real neuron this distinction would correspond to the case where one source of variability dominates over the other.

**Figure 1:**
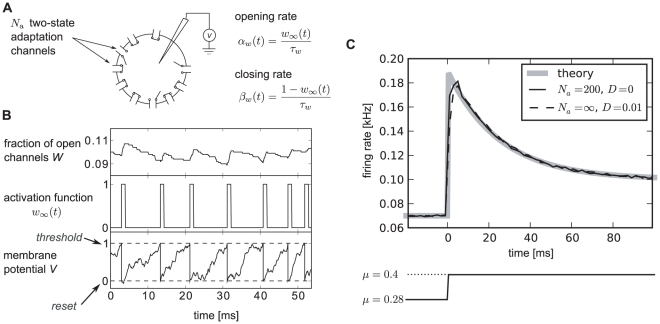
Integrate-and-fire dynamics with adaptation channels. **A** Channel model: a population of 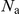 independent voltage-gated ion channels, which can be either in an open or a closed state, mediate an adaptation current through a neuron's membrane. **B** PIF model: Subthreshold dynamics of the membrane potential 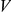 (bottom). The variable 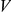 (measured in units of 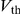) is reset to a value 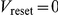 after crossing the threshold at 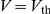. Action potentials are not generated explicitely. Instead, the effect of an action potential is captured by the activation function 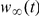, which is set to one in a short time window of 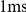 following each threshold crossing of the IF model (middle panel). The adaptation current is proportional to the fraction of open channels 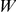 (top panel). The sample traces were obtained from a simulation of Eq. (35) with 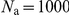 channels, white noise intensity 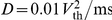, adaptation time constant 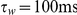, base current 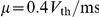 and maximal adaptation current 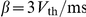. **C** The time-dependent firing rate (top) in response to a step stimulus (bottom) is independent of the source of noise (stochastic adaptation – solid line, deterministic adaptation plus white noise – dashed line). The gray line shows the theory given by Eq. (55).

In order to demonstrate the results on two different levels of complexity, we conducted both analytical investigations of a tractable integrate-and-fire model and a simulation study of a biophysically more realistic Hodgkin-Huxley-type neuron model. For the first model we chose the perfect (non-leaky) integrate-and-fire (PIF) model [2], [8]. This model represents a reasonable description in the suprathreshold firing regime, in which a neuron exhibits a stable limit cycle (tonic firing). The model was augmented with an inhibitory adaptation current mediated by a population of 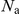 adaptation channels (Fig. 1A). For simplicity, we assumed binary channels that switch stochastically between an *open* and a *closed* state. The transition rates depend on the presence or absence of an action potential. This can be approximated by passing the membrane potential 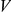 through a steady-state activation probability, 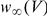, that attains values close to unity during action potentials, i.e. when the voltage exceeds the threshold, and is near zero for potentials below the firing threshold.

The PIF model, however, describes only the dynamics of the subthreshold voltage. In particular, it does not explicitely yield the suprathreshold time course of the action potential, but only the time instant of its onset (given by the threshold crossings). Nevertheless, the spike-induced activation of adaptation channels can be introduced in the PIF model. To this end, we approximated the activation function 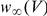 by a piecewise constant function of time, 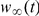, which attains unity for 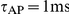 after the onset of each action potential and is zero otherwise (Fig. 1B, middle). A weak subthreshold activation of the adaptation current as observed for the M-current does not change the qualitative results of the paper (see Discussion). The time constant of the first-order channel kinetics was set by the parameter 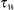.

Although our model aims at the stationary firing statistics, we would like to stress that it exhibits spike-frequency adaptation in the presence of time-varying stimuli. In particular, spike-frequency adaptation in response to a step stimulus is retained regardless of the considered noise source, channel numbers or approximations made during the theoretical analysis (Fig. 1C). This is a nice feature of the PIF model, for which the firing rate does not depend on the nature and magnitude of the noise. This allowed us to vary the noise properties without altering the adaptation properties.

### Diffusion approximation of channel noise

For the theoretical analysis the channel model describing the dynamics of each individual channel could be considerably simplified by a diffusion approximation. As shown in Methods, the dynamics of the finite population of adaptation channels can be described by (i) the deterministic adaptation current and (ii) additional Gaussian fluctuations with the same filter time as the adaptation dynamics. Together with our integrate-and-fire dynamics for the membrane potential 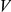 (Methods, Eq. (35)), we obtained a multi-dimensional Langevin model that approximates the IF model with stochastic ion channels (Methods, Eq. (20), (35)):
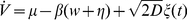
(1a)

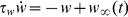
(1b)

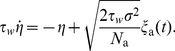
(1c)


**Figure 2:**
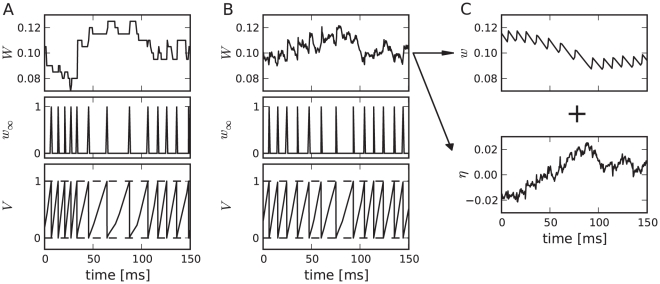
Diffusion approximation of adaptation current. **A** Sample traces of the integrate-and-fire dynamics with two-state adaptation channels, Eq. (20), (35) (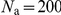 and 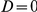). The fraction of open channels 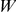 (top) exhibits discontinuous jumps with directions that depend on the presence or absence of a spike as given by the activation function 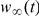 (middle panel). **B** Sample traces of the diffusion model, Eq. (1), with the same 1st and 2nd infinitesimal jump moments of 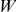 as in the channel model (**A**). **C** The fraction of open channels 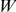 can be split into the deterministic part 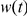, Eq. (1b), corresponding to 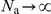, and an Ornstein-Uhlenbeck process 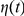, Eq. (1c), with a correlation time equal to the adaptation time constant (colored noise). The parameters are 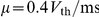, 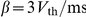, 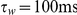.

Put differently, a finite population of slow adaptation channels (instead of an infinite population and hence a deterministic adaption dynamics) entails the presence of an additional noise 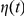 with a correlation time 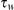 (time scale of the deterministic adaptation) and a variance which is inversely proportional to the number of channels. The membrane potential 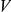 of the PIF model is thus driven by four processes: (i) the base current 
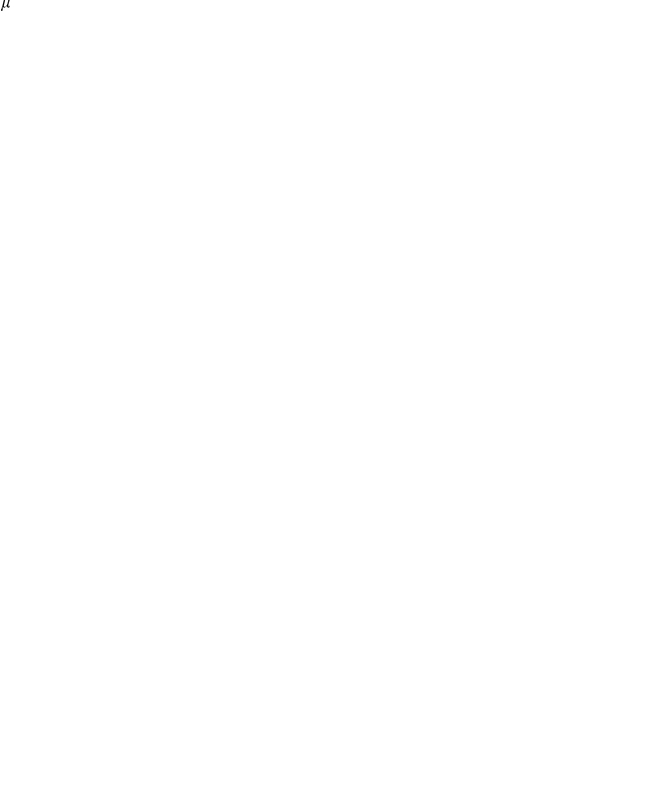
, (ii) the white current fluctuations 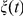 of intensity 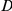 (representing an applied current stimulus, channel noise originating from fast sodium or delayed-rectifier potassium currents, or shot-noise synaptic background input), (iii) the slow noise 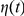 due to stochasticity of the adaptation dynamics, and (iv) the deterministic feedback of the neuron's spike train 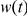 due to the deterministic part of the adaptation (see Fig. 2). In Eq. (1), the parameter 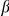 determines the strength of adaptation and 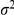 is set by the duration of the action potential 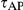 relative to the mean ISI (Methods, Eq. (41)).

To study the effect of the two different kinds of noise, we focused on two limit cases: In the limit of infinitely many channels, the adapting PIF model is only driven by white noise. In this case, Eq. (1) reads
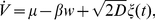
(2a)

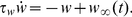
(2b)


We call this case *deterministic adaptation*. A dimensionality analysis shows that the ISI statistics are completely determined by the quantities 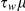 and 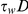 if one assumes 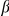 and 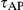 as constants (see Methods). Thus, decreasing 
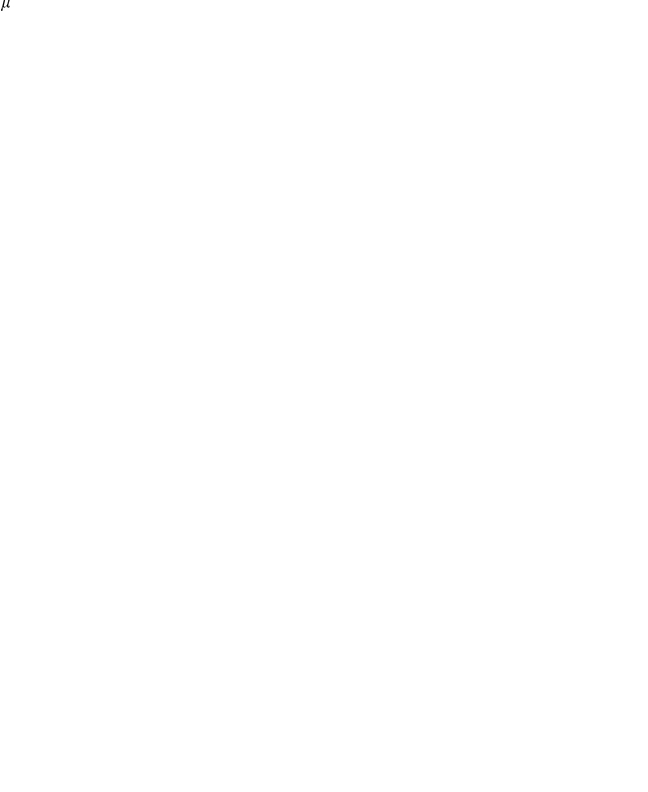
 and 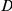 by some factor and simultaneously increasing 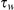 by the same factor (yielding a decreased firing rate 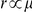) would, for instance, not alter the statistical properties of the model.

In the opposite limit, we considered only the stochasticity of the adaptation current but not the white noise. Setting 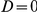 we obtain
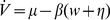
(3a)

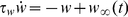
(3b)

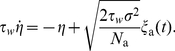
(3c)


We call this case (and the corresponding model based on individual adaptation channels) *stochastic adaptation*. As shown in Methods, this case is determined by the quantities 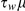 and 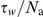 (assuming 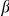 and 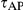 as constants). For instance, decreasing 
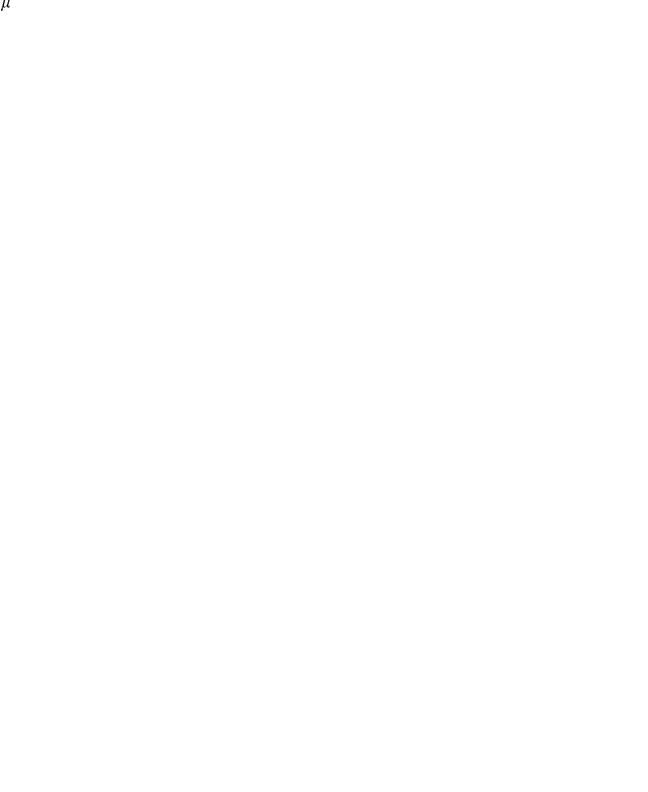
 and increasing 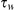 and 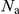 by the same factor (and thereby lowering the firing rate 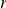) would again result in an equivalent model with the same statistical properties.

The fraction of open channels 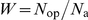 performs a random walk with discontinuous jumps. The direction of jumps depends on the presence of spikes, which in turn is affected by 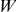 (Fig. 2A). The diffusion approximation of 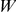 and the separation of deterministic and stochastic components are illustrated in Fig. 2B and 2C, respectively. Although the increments of the continuous diffusion process have the same (Gaussian) statistics as the original discontinuous process on a time interval larger than the mean ISI, the short-time statistics is rather different (Fig. 2A,B). Therefore, it is not obvious whether the diffusion approximation yields a good approximation to the ISI statistics, and in particular, how this approximation depends on the number of channels 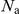 and the adaptation time constant 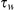. To clarify this issue, we performed both simulations based on individual channels (“channel model”) and simulations of Eq. (1) (“diffusion model”). It turned out, that the diffusion approximation yields a fairly accurate approximation for the shape of the ISI density, the coefficient of variation and the serial ISI correlations even for small channel populations. However, significant deviation were found for higher-order statistics like the skewness and kurtosis of the ISIH (see next section).

### Interspike interval statistics of the adapting PIF model

The calculation of the ISI statistics (histogram and serial correlations) of the PIF model with noise and spike-frequency adaptation is generally a hard theoretical problem. Here we put forward several novel approximations for the simple limit cases Eq. (2) and Eq. (3). For typical adaptation time constants that are much larger than the mean ISI we found the ISI histogram in the case of pure white noise (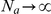, Eq. (2)) mapping the model to one without adaptation and renormalized base current 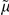 (Methods, Eq. (52)). This corresponds to a mean-adaptation approximation [18], [43]–[47], because the adaptation variable 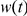 is time-averaged by the linear filter dynamics in Eq. (2b) for 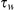 much large than the mean ISI (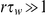). However, this approximation cannot account for ISI correlations, because any correlations between ISIs are eliminated in the limit 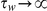 – in fact, the reduced model is a renewal model. For this reason, we developed a novel technique to calculate serial correlations for a PIF neuron with adaptation and white noise driving, which is valid for any time constant 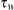 (see Methods).

In the opposite limit of only adaptation fluctuations (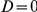, Eq. (3)), we could calculate analytically the ISI histogram, the skewness and kurtosis of ISIs as well as the ISI serial correlations by mapping the problem to one without an adaptation variable but a colored noise 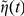 with renormalized parameters. Specifically, the IF dynamics for only adaptation channel noise reduces to
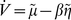
(4a)

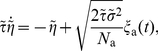
(4b)where the effective parameters are scaled by a common scaling factor:
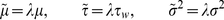
(5)with
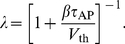
(6)


As before, a spike is fired whenever 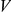 reaches 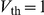, whereupon the voltage is reset to 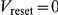. We call this model (Eq. (4)–(6)) the *colored noise approximation*. For the perfect integrate-and-fire model driven by a weak colored noise, i.e. for the model described by Eq. (4), analytical expressions for the ISI density and the serial correlation coefficient are known [48]. In addition to that, we derived novel analytical expressions for the skewness and kurtosis of the ISIs (see Methods).

Interestingly, the scaling factor in Eq. (6) has a concrete meaning in terms of spike-frequency adaptation: 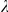 coincides with the degree of adaptation in response to a step increase of the base current (see Methods, Eq. (56)).

#### ISI density

**Figure 3:**
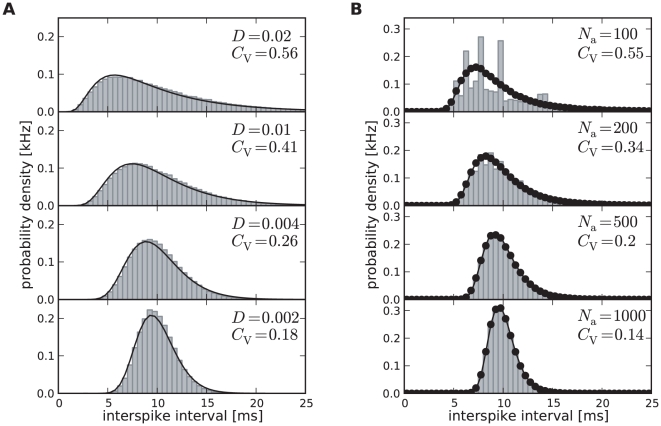
ISI histograms of a PIF neuron – theory vs. simulation. **A** ISI densities in the case of deterministic adaptation (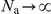) for different noise intensities 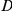. Gray bars show the histograms obtained from simulations of Eq. (2); solid lines display the mean-adaptation approximation, Eq. (64) (inverse Gaussian density). **B** ISI densities in the case of stochastic adaptation (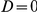) for different 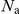 as indicated in the panels. The adaptation current was modeled either by the channel model (gray bars), Eq. (20), or by the diffusion model (circles), Eq. (1). The theory, Eq. (69), is displayed as a solid line. Parameters are chosen as in Fig. 2.

Fig. 3A shows ISI histograms (ISI densities) for the case of deterministic adaptation. We found, that the ISI densities can be well described by inverse Gaussian probability densities with mean 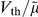 given by Eq. (64) (see Methods). In the opposite case of stochastic adaptation, the ISI variability solely depends on the number of slow adaptation channels (Fig. 3B). For a small channel population (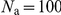) the discreteness of the adaptation 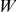 still appears in the ISIH as single peaks that cannot be averaged out. This is related to realizations of the channel noise for which the fraction of open channels does not change during the ISI; realizations for which the fraction changes at least once lead to the continuous part of the ISI density. In contrast, the diffusion model yields a purely continuous curve, that looks like a smoothed version of the ISIH of the model with channel noise. As 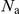 increases, the discrete peaks in the latter become more and more dense and insignificant, and the ISIH of the channel model is well approximated by the diffusion model. Furthermore, the theory for the colored noise approximation, Eq. (4), coincides well with the diffusion model, Eq. (1), and hence for sufficiently large 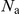 it also fits well the ISIH of the channel model.

**Figure 4:**
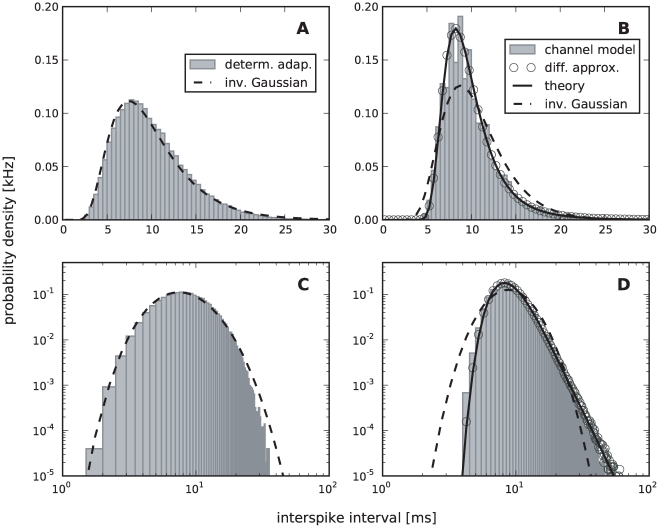
Comparison of ISIHs for deterministic vs. stochastic adaptation. **A** and **C** – The ISIH obtained from a simulation of the deterministic adaptation model, Eq. (2), with noise intensity 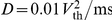 can be well described by an inverse Gaussian distribution (dashed line), Eq. (64). **B** and **D** – ISIH for the stochastic adaptation model with 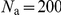 and 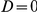. The channel model (gray bars) is more peaked than an inverse Gaussian distribution, Eq. (64), with the same mean and CV (dashed line). The ISIH of the diffusion model, (simulation of Eq. (3), circles) is well described by the colored noise approximation, Eq. (69), (solid line). Note the double logarithmic axis in **C** and **D** revealing the tail of the distribution. Other parameters as in Fig. 2.

One central claim of this paper is that ISI histograms of neurons, for which slow channel noise dominates the ISI variability, cannot be described by an inverse Gaussian (IG) distribution in contrast to cases where fast fluctuations dominate. We recall that the IG distribution yields the ISI histogram for a PIF model driven by white noise without any (deterministic or stochastic) adaptation, so *a priori* we cannot expect that this density fits any of the cases we consider here. However, as mentioned above, the ISI density can be captured by an IG for deterministic adaptation (Fig. 4A,C). In fact, the main effect of a slow adaptation is to reduce statically the mean input current which is reflected in our approximation by going from 
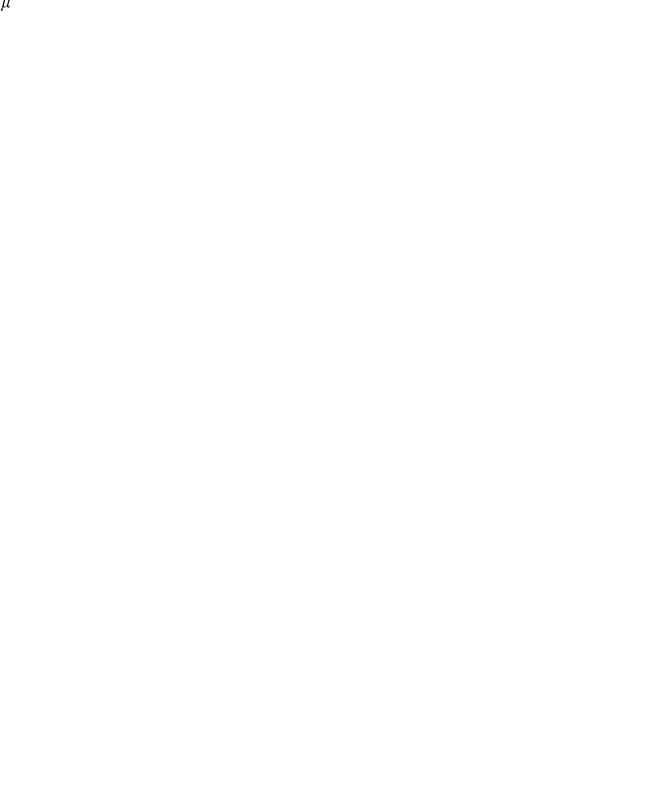
 to 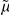. Slight deviations of the simulated ISI histogram from the IG can be seen for large intervals where the simulated density displays a stronger decay than the IG (Fig. 4C). With adaptation, large intervals are prevented because for large times (after the last spike) the inhibitory effect of the adaptation current subsides – a feature that is not present in the static approximation for the reduced base current which was made above. Nevertheless, the deviations are small and will be hardly visible when comparing the IG density to the histogram of limited experimental data sets.

By contrast, the ISI histogram in the case of stochastic adaptation as illustrated in Fig. 4B and D, possesses a much stronger peak and decays slower at large ISIs than the IG with the same mean and variance of the ISI; comparison to the IG density that has the same mean and mode or the same mode and CV gave comparably bad fits (data not shown). Instead, the colored noise approximation as outline above, describes the simulation data fairly well.

**Figure 5:**
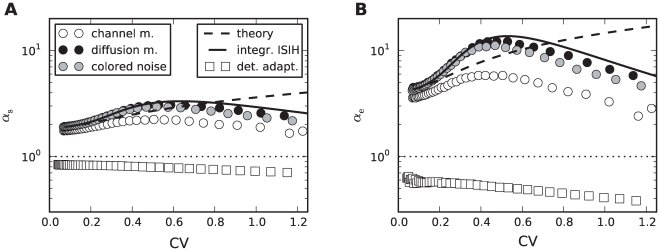
Shape parameters of the ISIH for deterministic and stochastic adaptation. **A** Rescaled skewness 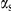 for deterministic adaptation (white squares) and for stochastic adaptation (channel model – white circles, diffusion model – black circles, colored noise approximation – gray circles). Different CVs were obtained by varying 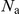 or 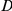. The dashed line depicts the theoretical curve, Eq. (7), and the solid line depicts the semi-analytical result obtained from the moments of the ISI density, Eq. (69), using numerical integration. **B** The corresponding plot for the rescaled kurtosis 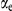. The adaptation time constant was 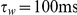. All other parameters as in Fig. 2.

This suggests that both cases – deterministic and stochastic adaptation – might be distinguishable from the shape of the ISI histograms even if mean and CV of the ISIs are comparable as for the data in Fig. 4. To this end, we introduced new measures 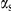 and 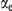 based on the skewness and the kurtosis (excess) of the ISI distribution that are exactly unity for an IG distribution (see Methods, Eq. (61) and (62)). Indeed, Fig. 5 reveals that deterministic and stochastic adaptation are well separated with respect to the rescaled skewness and kurtosis 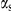 and 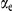. In particular, these quantities are clearly larger than unity for stochastic adaptation meaning that the ISI density is more skewed and more peaked compared to an IG, which confirms our previous observations. On the other hand, for deterministic adaptation, 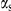 and 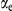 are smaller than unity in accordance with our previous observations that the tail of the ISI density decays slightly faster than an IG density.

**Figure 6:**
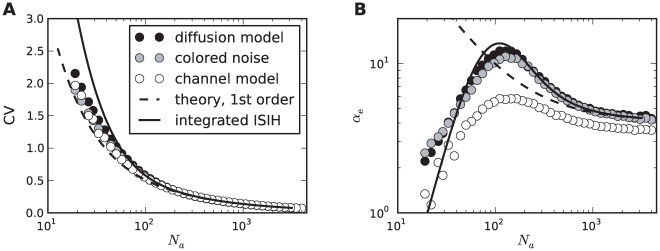
Comparison of diffusion and channel model. **A** The coefficient of variation as a function of the number of adaptation channels 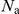 for the diffusion model (black circles, Eq. (1)), the channel model (white circles, Eq. (20)) and the colored noise approximation (grey circles, Eq. (4)). The dashed line depicts the theoretical curve Eq. (71) and the solid line depicts the semi-analytical result obtained from the moments of the ISI density, Eq. (69), using numerical integration. **B** The corresponding curves for the rescaled kurtosis 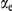. The dashed line represents the theory given by Eq. (114). The time scale separation was 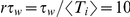. Parameters as in Fig. 2.

The rescaled kurtosis reveals also differences between the channel and the diffusion model. In Fig. 6A, the CV still matches almost perfectly for both models even at extremely small channel numbers, where the Gaussian approximation is expected to fail. This is also remarkable in the light of the discrete structure of the ISIH for small channel numbers (cf. Fig. 3 for 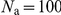). However, in Fig. 6B it becomes apparent that the two models differ with respect to higher-order measures as 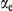; for increasing numbers of channels the differences decrease.

Fig. 5 and 6 also support the colored noise approximation, which describes the diffusion model quite accurately. This suggests, that the heavy-tailed and pronouncedly peaked ISIH in the case of stochastic adaptation can be simply explained by the effect of a long-correlated, colored noise. It is known that for the related leaky IF model such correlations result in ISIHs with a large kurtosis [49]. To examine the role of long-range temporal correlations in shaping the ISI density we analyzed the dependence of the ISI statistics on the time-scale separation between adaptation time constant and mean ISI. This can be quantified by the ratio 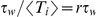, where 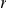 and 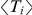 denote the stationary firing rate and the mean ISI, respectively.

In the case of stochastic adaptation, we obtained analytical expressions for 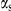 and 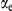 using the colored noise approximation for weak noise (see Methods). For the following discussion, it suffices to consider the zeroth order of the weak-noise expansion, which is given by
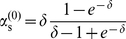
(7)and
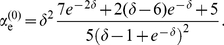
(8)


These expressions only depend on the non-dimensional parameter 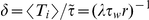, i.e. on the product of rescaled adaptation time constant and firing rate. From Eq. (7) and (8), it can be shown that 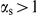 and 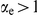 for 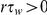 and that both quantities converge to unity in the limit 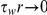. For 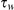 much larger than the mean ISI, i.e. when 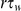 is large, the leading orders saturate at 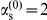 and 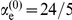.

**Figure 7:**
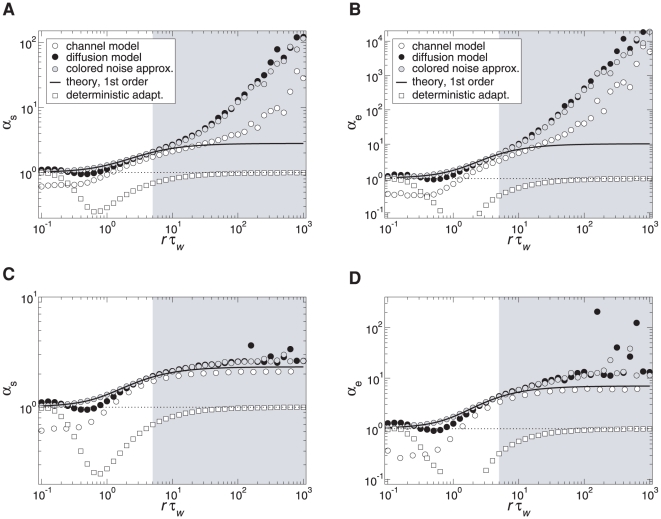
Shape parameters of the ISIH as a function of the time scale separation. **A** Rescaled skewness 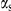 and **B** rescaled kurtosis 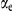 for 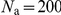 channels (corresponding 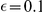) for stochastic adaptation (circles) and 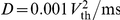 for deterministic adaptation (squares). Theory Eq. (113) and (114) is displayed by the solid line, the line 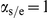 is indicated by a dotted line. **C** and **D** corresponding plots for 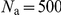 channels (corresponding to 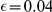) and 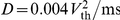. 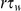 was varied by changing 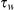 at a fixed 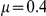 (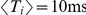), all other parameters as in Fig. 2.

These predictions are confirmed by simulations using different 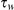 at a fixed value of 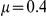 (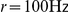, Fig. 7), thereby varying the time scale separation 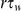 (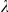 remains constant). In particular, both rescaled skewness and kurtosis are larger than unity and increase strongly at moderate fluctuations (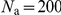, Fig. 7A,B). The increase is more pronounced for the diffusion model compared to the channel model. At large 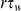 the simulation data deviate from the first-order approximation, because higher-order terms in the small noise expansion cannot be neglected. The agreement becomes better when the number of channels becomes larger (see Fig. 7C,D for 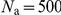). These observations were qualitatively confirmed by corresponding simulations at a smaller base current (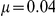) leading to a lower firing rate of 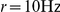 (data not shown). In particular, quantitatively similar curves were obtained when at 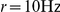 the number of channels was increased to 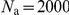 in order to maintain the same effective noise level. It should be noted, that in the case of low firing rates the weak-noise expansion might become infeasible if the number of channels is too small. This is, for example, the case for 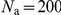 at 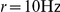, for which the small parameter 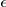 (Eq. (68)) becomes larger than unity.

For deterministic adaptation 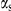 and 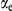 approach unity for 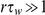 as predicted by the mean-adaptation approximation, for which the ISIH is given by the IG (Eq. (64)). In the opposite limit of small 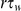 the parameters 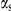 and 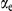 also approach unity. This is intuitively clear, because for 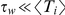 the adaptation 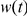 decays quickly to zero after each spike. Hence, the base current is almost always equal to 
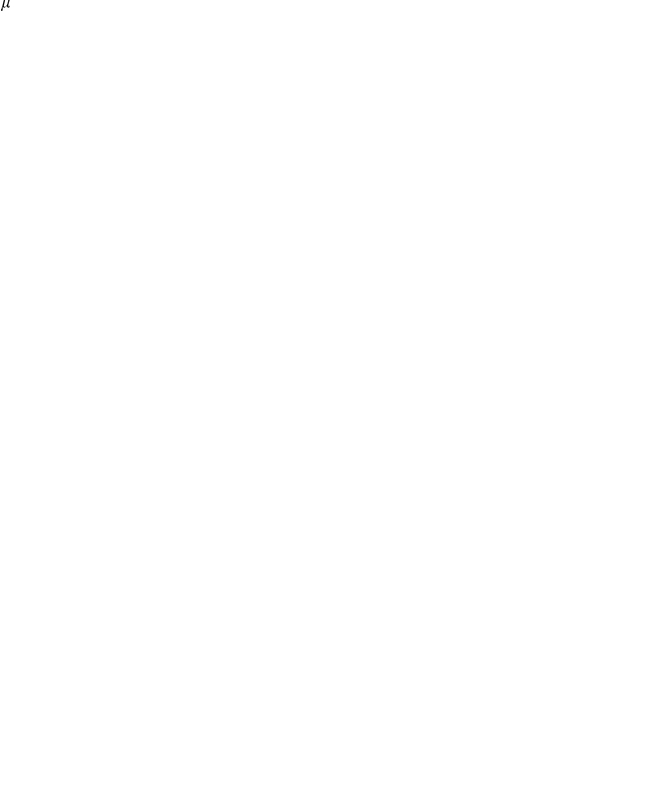
 except for a very short period after a spike where the driving is 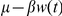. Put differently, the dynamics can be approximated by a PIF model with a constant driving 
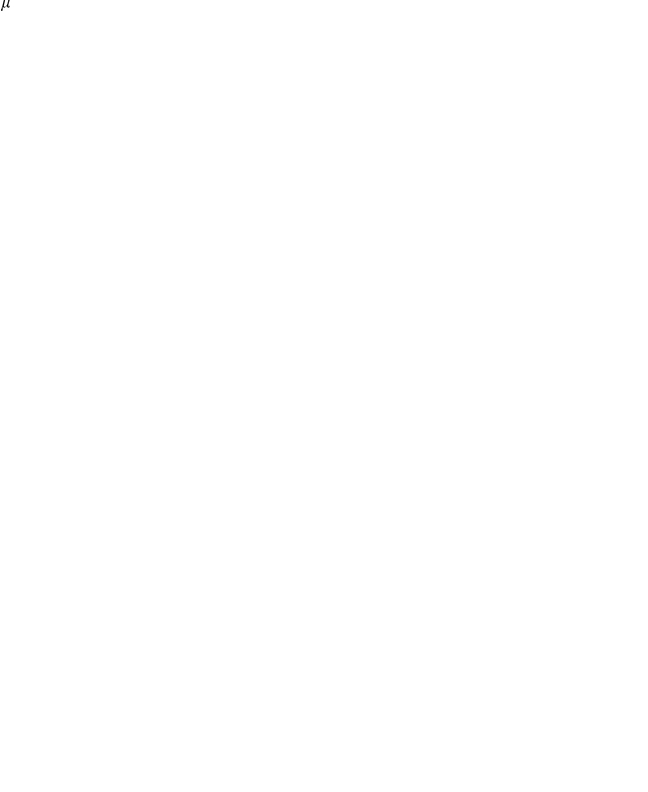
 and an effective reset value 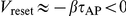. In this case, the ISIs are again distributed according to the IG statistics.

In the intermediate range, where the time scale of the adaptation is of the same order as the mean ISI, a pronounced minimum of 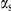 and 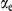 is observed in the case of deterministic adaptation. This is due to the decay of adaptation at such a rate that large ISIs are suppressed. As a consequence, the tail of the ISIH decays faster and the ISIH becomes less skewed compared to an IG. The same qualitative behavior was verified in simulations at a lower firing rate 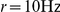 (data not shown).

#### ISI correlations

Another clear distinction between stochastic and deterministic adaptation is revealed by the correlations between ISIs. In several modeling studies it has been found that negative feedback mechanisms like adaptation currents in the presence of white noise give rise to negative correlations between adjacent ISIs [28], [29]. However, a theoretical explanation of this effect has not been provided yet. Therefore we developed a theory based on the dynamics close to the deterministic limit cycle of the adaptation dynamics (see Methods). This dynamics can account well for the correlations between ISIs in the case of deterministic adaptation. Specifically, the serial correlation coefficient (SCC) for two ISIs with lag 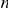 (see Eq. (63)) is given by the geometric sequence
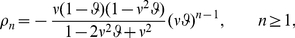
(9)where
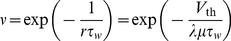
(10)and
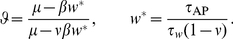
(11)


**Figure 8:**
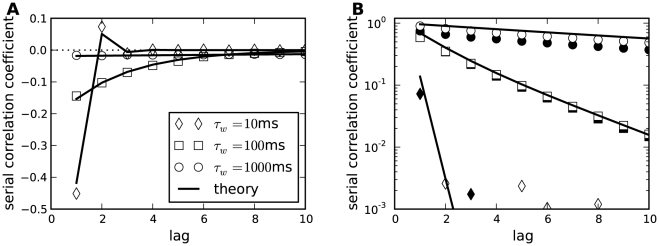
Serial correlation coefficient as a function of the lag between ISIs. **A** The case of deterministic adaptation with 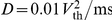 for different values of the time constant 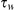 (as indicated in the legend). The theoretical curves, Eq. (9), are depicted by solid lines; the zero baseline is indicated by a dotted line. **B** The case of stochastic adaptation with 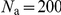 for different values of the time constant 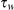 (as in **A**). The channel model, Eq. (20), is represented by white symbols, the diffusion approximation (Eq. (1)) is represented by black symbols. The theory based on the colored noise approximation, Eq. (4), is depicted by a solid line. Other parameters as in Fig. 2.

Noting that 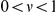 and 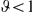, we find that the prefactor in front of the term 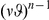 is negative. Thus, correlations at odd lags are always negative, whereas ISIs with even lag are anti-correlated only if 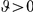. If on the other hand 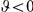, ISIs with even lags exhibit a positive SCC, giving rise to oscillations of 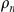. Both cases, purely negative correlations with an exponential decay and oscillating correlations, are indeed observed for deterministic adaptation (Fig. 8A). The slight deviation of the theoretical prediction is due to the short rise phase of 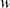 following each spike in the simulations instead of the instantaneous increase of 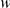 assumed in the derivation of Eq. (9).

In striking contrast, the case of stochastic adaptation yields positive ISI correlations with a slow exponential decay (Fig. 8B) as predicted by the theory, Eq. (72). From the formula it becomes evident, that the decay constant is to first-order given by the ratio 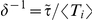 of effective correlation time 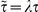 and mean ISI. The good agreement of the colored noise theory suggests, that adaptation noise effectively acts as a colored noise that slowly modulates the ISIs. It is known, that systems with a slow stochastic driving exhibit positive ISI correlations [48]–[50]. In fact, in the absence of additional fast fluctuations the ISIs are strongly correlated with the slow noise, which retains a memory of previous ISIs. For instance, if 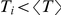 due to a large, positive fluctuation of 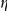, this will on average cause a likewise small 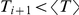, because the slow dynamics of 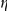 tends to persist at positive values in the course of several subsequent ISIs.

**Figure 9:**
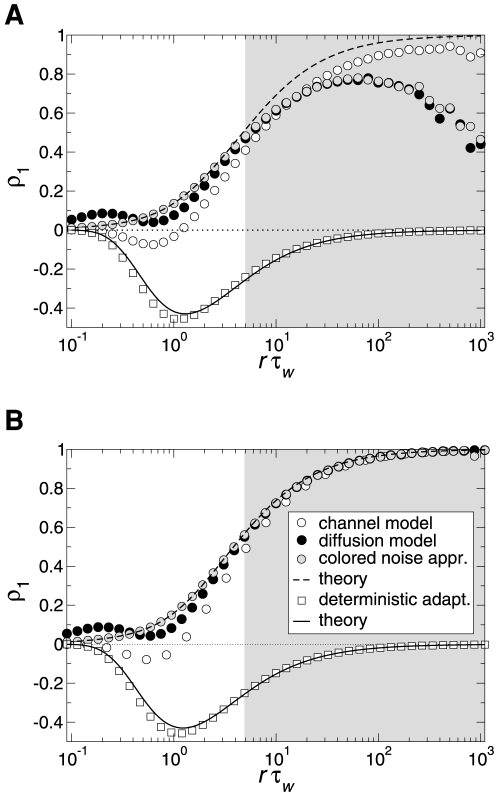
Serial correlation coefficient at lag 1 as a function of the time scale separation. **A** Serial correlation coefficient 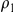 in the case of 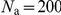 channels (corresponding to 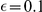) for stochastic adaptation (circles, Eq. (3)) and 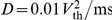 for deterministic adaptation (squares, Eq. (2)). Theoretical curves for stochastic adaptation, Eq. (72), and deterministic adaptation, Eq. (9), are displayed by a dashed line and a solid line, respectively. The zero baseline is indicated by a dotted line. **B** shows the corresponding plot for 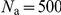 channels (corresponding to 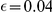) and 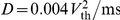. The gray-shaded region marks the relevant range for spike-frequency adaptation. 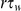 was varied by changing 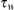 at fixed 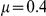 (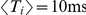), all other parameters as in Fig. 2.

ISI correlations are strongly governed by the time scales in the system. We therefore investigated the role of the time scale separation parameter 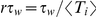 on the serial correlations of adjacent ISIs (Fig. 9). For deterministic adaptation we found that adjacent ISIs become most anti-correlated at a finite value of 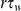 close to unity. In the limits 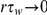 and 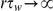, however, 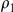 vanishes as predicted by the theory and as observed in previous studies [29], [34]. This is intuitively clear, because in the first limit the adaptation variable 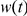 cannot accumulate by subsequent spikes, and hence no memory of previous ISIs is retained; in the latter limit because 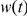 converges to its (constant) mean value for 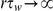. Interestingly, the ISI correlations seem to be almost independent from the noise intensity 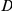 if 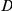 is not too large (compare Fig. 9A and B for two different values of 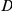). This insensitivity of the correlation coefficient to the noise intensity could be anticipated from the analytical theory (see Methods), in which the noise dependent term cancels out in the ratio of ISI covariance and ISI variance.

For stochastic adaptation, the positive correlations become strongest for 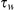 much larger than the mean ISI (Fig. 9), i.e. 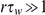. The channel and diffusion model agree generally quite well, except for very small and very large 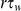. In the latter case, the Gaussian approximation becomes worse, because the opening and closing events of the channels are extremely rare. The expected number of channel transitions in a time window of length 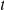 is 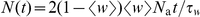 with 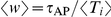. For instance, taking the extreme case 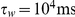 at the standard parameters 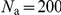, 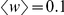 one would on average observe only 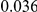 transitions on the time scale of a single ISI (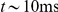). On this time scale the fraction of open channels can hardly be approximated by a Gaussian process. As expected, we obtained a better agreement between channel and diffusion model at large 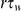 by increasing the number of channels (Fig. 9B). The decrease of 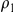 for the diffusion model and the colored noise approximation at very large 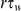 might be due to the fact that the ISI variance grows faster with 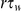 than the covariance 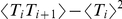, thus the correlation coefficient is suppressed by the variance. A similar effect has been observed for the LIF model [49].

#### Mixed case of fast and slow noises

So far, we found that the two limit cases of the adapting PIF model can be well distinguished by the values of the shape parameters 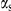 and 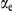 relative to unity and the correlation coefficient 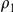 relative to zero. Do these quantities also allow for an unambiguous distinction of the dominating source of noise in the more realistic case where both kinds of noise are present? To answer this question, we performed simulations of the adapting PIF model for a fixed intensity of the white noise (“fast fluctuations”) but different sizes of the population of adaptation channels. Thereby, we could vary the ratio of the two different types of noise. Note, that the mean adaptation current is kept constant in our setting. This can be realized by scaling the single channel conductance or the membrane area with 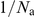 (see Methods).

**Figure 10:**
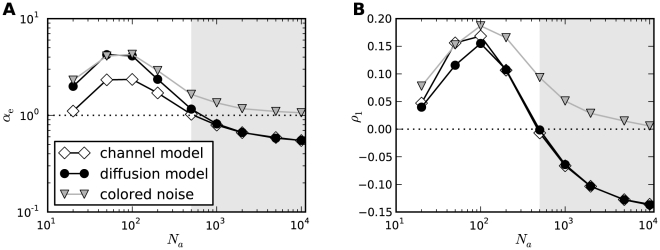
ISI statistics of the PIF model in the presence of both stochastic adaptation and white noise. For a fixed level of white noise (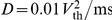) the number of adaptation channels 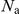 was varied. For a small channel population slow channel noise dominates (white region), whereas for a large population the fast fluctuations dominate (gray-shaded region). **A** Rescaled kurtosis 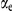 for the channel model (white diamonds) and the diffusion model (black circles). The gray symbols display simulations where the adaptation was replaced by an effective colored noise as before but with the additional white noise input. The case of an inverse Gaussian is indicated by the dotted line. **B** Corresponding serial correlation coefficient at lag one. The zero line is indicated by the dotted line. The adaptation time constant was chosen as 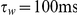, other parameters as in Fig. 2.

For small channel numbers, i.e. large channel noise, we observed both large values of the rescaled kurtosis 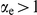 and a positive serial correlation coefficient of adjacent ISIs (Figs. 10A,B, white region) indicating the strong impact of the colored noise effect. As expected, at the other end of large channel population sizes the pure white noise case could be recovered. In between, we found a critical channel number at which both the rescaled kurtosis crossed the line 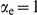 and the serial correlation coefficient changed its sign. This simultaneous change suggests, that below the critical channel number the ISI statistics was dominated by slow adaptation channel noise, whereas above this critical size it was dominated by the white noise input (gray-shaded region).

### Effects of a stochastic adaptation current on the ISI statistics of a Hodgkin-Huxley type model

**Figure 11:**
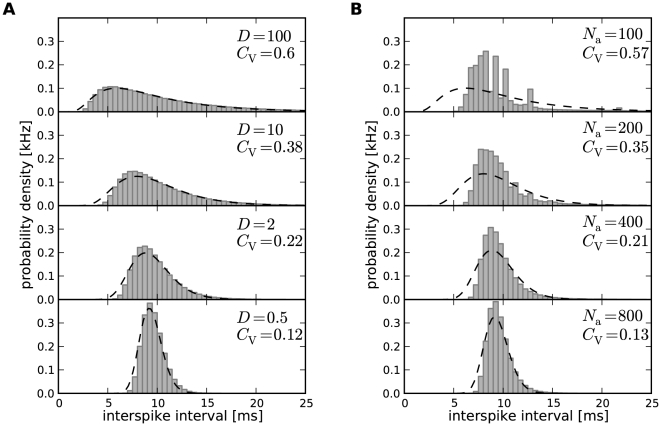
ISI histograms of the Traub-Miles model – deterministic vs. stochastic adaptation. **A** The ISI densities of the Traub-Miles neuron model with a deterministic M-type adaptation current (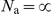) and white noise driving (Eq. (115) – gray bars) is shown along with an inverse Gaussian (Eq. (64)) with the same mean and CV (dashed lines). To keep the firing rate at about 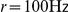 the external driving current was adjusted from top to bottom according to 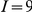, 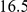, 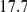, 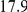 (in 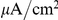). Noise intensity 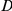 in units of 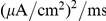. **B** The ISI densities of the Traub-Miles model in the presence of a stochastic M-type adaptation current (Eq. (116) – gray bars) is shown along with an inverse Gaussian (Eq. (64)) with the same mean and CV (dashed line). Here, the external driving current was in all cases 
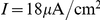
.

We investigated whether our theoretical predictions based on a simple integrate-and-fire model are robust with respect to a more detailed model of the Hodgkin-Huxley type. To this end, we performed simulations of the conductance-based Traub-Miles model with a M-type adaptation current [51]. As in the previous model we separately considered the two cases of white noise input and a slow M-type channel noise to get an intuition of the individual effects on the ISI statistics. Fig. 11 demonstrates that the ISI histograms show essentially the same features as in the PIF model: in the case of white noise input the shape of the ISIH could be well approximated by an inverse Gaussian distribution which was uniquely determined by the firing rate and the CV. In the case of a stochastic M-type current there is a strong disagreement between the ISIH and an inverse Gaussian with the same rate and CV. In particular, ISIHs exhibited again a sharper peak compared to the relatively broad inverse Gaussian.

**Figure 12:**
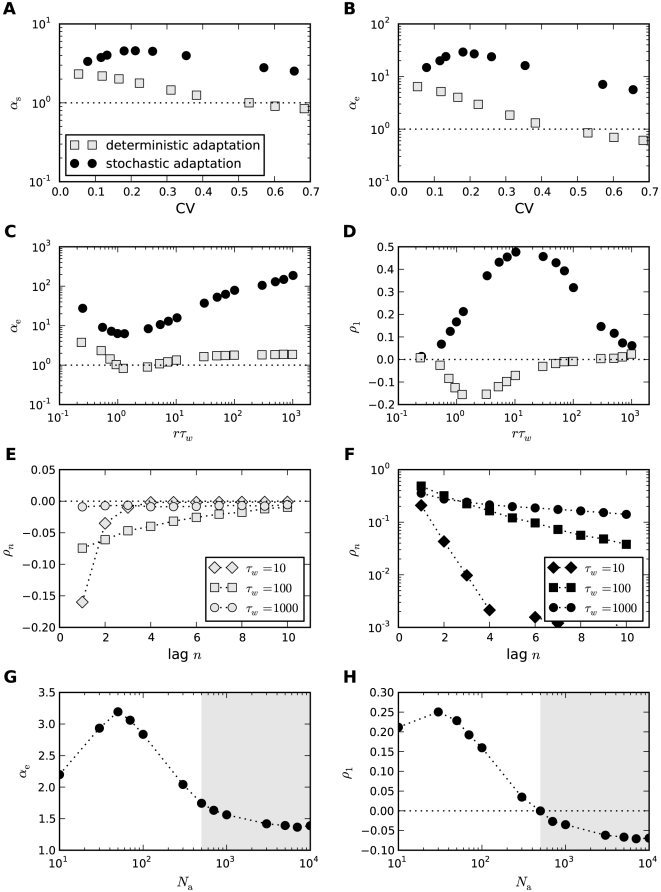
Comparison of the ISI statistics of the Traub-Miles model – deterministic vs. stochastic adaptation. **A** Rescaled skewness 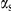 (Eq. (61)) and **B** rescaled kurtosis 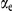 (Eq. (62)) as a function of the coefficient of variation (CV). For stochastic adaptation (Eq. (116), 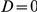 – black circles) the number of channels was varied from 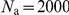 to 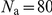; for deterministic adaptation (Eq. (115), 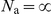 – gray squares), the noise intensity was varied from 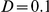 to 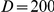. The corresponding inverse Gaussian statistics (Eq. (64)) is indicated by the dotted line. **C, D** show the rescaled kurtosis and the serial correlation coefficient (Eq. (63)) at lag 1 as a function of the time scale separation 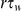. Stochastic adaptation (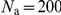) and deterministic adaptation (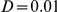) are marked as in **A,B**. **E,F** The serial correlation coefficient 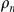 as a function of the lag 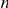 for different time constants 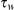 in 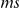 as indicated (**E** deterministic adaptation, **F** stochastic adaptation; 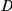 and 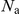 as in **C,D**). **G** The rescaled kurtosis 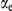 in the mixed case at a fixed amount of white noise (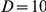) and varying channel numbers 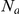. **H** The corresponding values of the serial correlation coefficient at lag one. The intersection of the 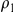 curve with the zero line (dotted line) defines the adaptation-noise dominated regime (white region) and the white-noise dominated regime (gray-shaded region). The units of the noise intensities are 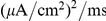. For stochastic adaptation 
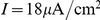
. For deterministic adaptation 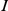 was adjusted to result in a firing rate at around 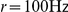. For 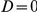 the current 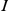 was 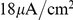. With increasing noise intensity 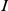 decreased to 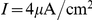 for 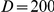.

The different ISI statistics for the case of deterministic and stochastic adaptation are analyzed more closely in Fig. 12. As in the PIF model (cf. Fig. 5) the rescaled skewness and kurtosis are significantly smaller for white noise than for adaptation noise in a wide range of CVs (Fig. 12A,B). This is in accordance with the pronounced peak of the ISIH in the case of stochastic adaptation (Fig. 11B). However, the values are not strictly separated by 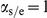 as in the PIF model. This discrepancy is not surprising, given that the Traub-Miles dynamics with constant input and white noise driving does not exactly yield an inverse Gaussian ISI density but only an approximate one. Importantly, however, the rescaled kurtosis 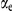 quickly saturates at a finite value in the large 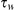 limit (albeit not at unity, Fig. 12C). This is markedly different from the case of stochastic adaptation. In this case, the rescaled kurtosis increases strongly as it was observed for the PIF model. In a similar manner, the rescaled skewness also showed this distinct behavior for stochastic vs. deterministic adaptation, although the increase of the rescaled skewness was not as strong as for the rescaled kurtosis (data not shown).

A clear distinction between both cases appears in the serial correlations of ISIs (Fig. 12D). Similar as in the PIF model, the case of deterministic adaptation is characterized by negative ISI correlations at lag one, which are strongest at an intermediate time scale 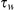. Furthermore, the case of stochastic adaptation exhibits positive correlation coefficients 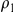, which show a maximum at an intermediate value of 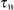. This is also in line with the PIF model. The correlations decay rapidly with the lag for deterministic adaptation (Fig. 12E) and decay exponentially for stochastic adaptation (Fig. 12F). As in the PIF model, the exponential decay is slower for large time constants 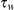.

Finally, we inspected the case in which both white noise and slow adaptation noise is present (Fig. 12G,H). As in Fig. 10 for the PIF model, we fixed the noise intensity of the white noise and varied the number of adaptation channels 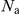. In the Traub-Miles model one finds qualitatively similar curves as in the PIF model. In particular, the serial correlation coefficient at lag one, shows a transition from positive to negative ISI correlations at a certain number of adaptation channels (Fig. 12H). As for the PIF model, this value can be used to define two regimes – one dominated by adaptation noise (white region) and another one dominated by white noise (gray-shaded region). In the adaptation-noise dominated regime the parameter 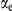 is larger than in the white-noise dominated regime (Fig. 12G).

The observation that key features of the ISI statistics in the presence of a stochastic adaptation current seem to be conserved across different models suggests a common mechanism underlying these features. As we saw, this mechanism is based upon the fact that a stochastic adaptation current can be effectively described by an *independent* colored noise. The long-range temporal correlations of this noise naturally yield positive ISI correlations and a slow modulation of the instantaneous spiking frequency. The latter typically involves a large kurtosis due to the increased accumulation of both short and long ISIs. A significant amount of colored noise can effect the kurtosis and the ISI correlations so strongly, that details of the spike generation seem to be of minor importance. Thus, it becomes plausible that the spiking statistics of a rather complex neuron model could be explained by a simple integrate-and-fire model including a stochastic adaptation current.

## Discussion

In this paper, we have studied how a noisy adaptation current shapes the ISI histogram and the correlations between ISIs. In particular, we have compared the case of pure stochastic adaptation with the case of a deterministic adaptation current and an additional white noise current. Using both a perfect IF model that is amenable to analytical calculations and a more detailed Hodgkin-Huxley type model, we found large differences in the ISI statistics depending on whether noise was mediated by the adaptation current or originated from other noise sources with fast dynamics. As regards the ISI histogram, stochasticity in the adaptation leads to pronounced peaks and a heavy tail compared to the case of deterministic adaptation, for which the ISI density is close to an inverse Gaussian. To quantify the shape of ISI histograms we proposed two measures that allow for a simple comparison with an inverse Gaussian probability density that has the same mean and variance. The first one is a rescaled skewness (involving the third ISI cumulant); the second is a rescaled kurtosis (involving the fourth ISI cumulant). Both quantities possess the property that they assume unity for an inverse Gaussian distribution. If they are larger than unity as in the case of stochastic adaptation the ISI density is more skewed or respectively has a sharper peak and a heavier tail than an inverse Gaussian density with the same variance. If these measures are smaller than one, the ISI histogram tends to be more Gaussian like. Most strikingly, we found that for a stochastic adaptation current the rescaled skewness and kurtosis strongly increase when the time scale separation of adaptation and spiking becomes large (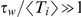). By contrast, for a deterministic adaptation current the rescaled kurtosis saturates close to one in this limit.

Another pronounced difference arises in the ISI correlations. For a deterministic adaptation current and a white noise driving one observes short-range anti-correlations between ISIs as reported previously (e.g. [29]). In contrast, with slow adaptation noise ISIs exhibit long-range positive correlations. In the presence of both types of noise, the serial correlation coefficient changes continuously from positive to negative values when the ratio of white noise to adaptation noise is increased. The two domains might be useful in determining the dominating source of noise from a neural spike train.

Interestingly, the perfect integrate-and-fire model augmented with an adaptation mechanism predicted all the features seen in the spiking statistics of the Traub-Miles model with stochastic adaptation and/or white noise input. This indicates the generality and robustness of our findings. It also justified the use of the adapting PIF model as a minimal model for a repetitive firing neuron with spike-frequency adaptation. It seems, that in the suprathreshold regime the details of the spike generator are of minor importance compared to the influence of adaptation and slow noise.

By means of the PIF model one can theoretically understand the underlying mechanism leading to the large kurtosis and the positive ISI correlations in the case of stochastic adaptation. This rests upon the fact that slow adaptation noise effectively acts as an independent colored noise with a large correlation time. One can think of the colored noise as a slow external process that slowly modulates the instantaneous firing rate or, equivalently, slowly changes the ISIs in the sequence. Such a sequence of many short ISIs in a row and a few long ISIs gives rise to a large skewness and kurtosis and positive serial correlations. In previous works, slow processes which cause positive ISI correlations were often assumed to originate in the external stimulus [49], [50], [52]. Here, we have shown that an intrinsic process, i.e. the fluctuations associated with the stochasticity of adaptation, yields likewise positive ISI correlations. Our finding also provides an alternative explanation of positive ISI correlations in experimental studies [30], [53]. Moreover, *in vivo* recordings from a looming-sensitive interneuron in the locust optic lobe have revealed both positive correlations at large firing rates and negative correlations at low firing rates [23]. Because this neuron exhibits pronounced spike-frequency adaptation an intriguingly simple explanation for these observations would be the presence of both fast noise and stochastic adaptation (corresponding to our mixed case). In this case, a large firing rate could indeed lead to a large effective correlation time of the noise associated to the adaptation mechanism and thus to positive ISI correlations.

Spike-frequency adaptation has been ascribed to different mechanisms (see e.g. [18]), involving for instance, calcium-dependent potassium currents 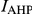 [42], slow voltage-dependent M-type currents 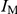 [41], [42] and slow recovery from inactivation of sodium currents [54]. Here, we chose the M-current as an example to illustrate the emergence of noise in the adaptation mechanism. In this specific case, it was the finite number of M-type potassium channels that gave rise to slow channel noise. For the other commonly studied adaptation mechanism, the 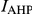 [18], , we have to deal with two possible sources of noise: the finite number of potassium channels 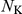 and fluctuations of the local 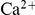 concentration 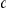. Proceeding in a similar fashion as for 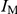, we would obtain 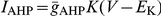, with the fraction 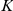 of open potassium channels, obeying
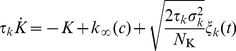
(12)

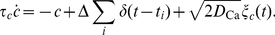
(13)


Here, the Gaussian white noises 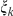 and 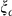 approximately represent the channel noise and the concentration fluctuations due to stochastic removal of calcium, respectively. The calcium gating is characterized by the steady-state activation 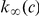. For simplicity, the increase of calcium 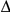 caused by an action potential is assumed to be deterministic. Importantly, however, the channel dynamics is fast compared to the slow removal of calcium, i.e. 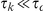. Following [18] the open probability of the potassium channels 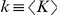 adiabatically adjusts to 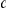 (i.e. 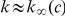) and the relationship is roughly linear (i.e. 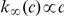). Thus, we have 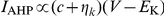, where the “channel noise” 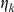 possesses a correlation time 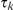. If this correlation time is much smaller than the mean ISI, the channel noise can be approximately treated as a white noise. But this means, that a PIF neuron with a calcium-dependent 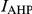 instead of 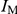 can likewise be approximated by Eq. (1): the fast channel noise can be included into the white noise term 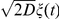 and the slow fluctuations of the calcium concentration assume the role of the slow adaptation noise 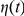. Approximating 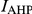 again by a voltage-independent current, the PIF model with 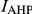 would read
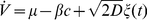
(14)

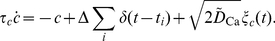
(15)


These equations can indeed be put into the form of Eq. (1) by splitting the deterministic and the noise part of 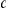. This illustrates that the main results derived in this paper are not specific to a certain adaptation current, but apply quite generally to any noise associated to the slow dynamics of adaptation.

The adaptation currents 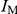 and 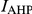 have been distinguished with respect to their ability to synchronize coupled neurons [51] and regarding the influence on neural coding [55]. The difference consists in whether the current is activated solely by spikes as in the case of 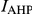 or whether it is also activated by subthreshold voltages as for 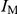. For the sake of clarity, we have set the activation function 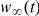 of the M-type adaptation current in the PIF model equal to zero at subthreshold voltages, i.e. between spikes (Eq. (25)). Thus, the adaptation current in the PIF model, unlike the M-current, was only activated during action potentials. It is, however, easy to show that the results of this paper are unchanged if subthreshold activation is allowed. For simplicity, let us consider the extension that in-between spikes the steady-state activation function 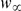 is equal to the value 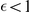, i.e. instead of Eq. (25), (26) (see Methods) we have
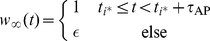
(16)

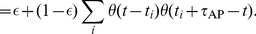
(17)


This only increases the mean adaptation to 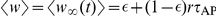 (cf. Eq. (37)). Similarly, the variance changes according to 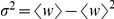 (cf. Eq. (40) in Methods). As a result, the effective base current is now given by
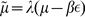
(18)with the new scaling factor
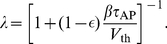
(19)


The colored noise approximation can be carried out in an analogous manner yielding the same result Eq. (4) (again with 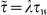, 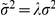, but the new scaling factor 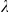, Eq. (19)). Thus, it can be expected that in the presence of subthreshold activation of 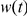 the colored noise effect (i.e. pronounced peak of ISIH, positive ISI correlations) in the case of stochastic adaptation is preserved. Furthermore, 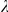 still serves as the degree of adaptation, i.e. the ratio of steady-state to initial gain when a step current is applied.

The analytical calculation of *higher-order* statistics in the presence of adaptation is a fundamental theoretical problem, which has been largely ignored so far (for a recent exception see [37]). Here, we succeeded to provide explicit expressions for the ISI histogram and their serial correlations for both white noise driving and noise in the adaptation dynamics. This was achieved by analyzing a spike generator and a channel model that are as simple as possible. There are certainly a lot of details that can be modeled in a more realistic way. For instance, it is known that the M-channel kinetics is governed by several time scales and more than two internal states [56]. Furthermore, channels might not be strictly independent, but channel clusters might exhibit cooperative behavior [57]. The latter case, would actually increase the level of channel noise compared to the case of independent channels, i.e. cooperativity would contribute to stochastic adaptation.

For many neurons physiological details like the number of ion channels are hard to obtain directly from experiments. Instead given the spike train statistics of a neuron, our study could be useful to judge whether M-channels or other adaptation mechanisms could potentially contribute to the neuronal variability. Furthermore, it is not impossible to think of experiments, in which the number of adaptation channels is reduced (e.g. by the mild application of a channel blocker) and thus the effects of stochastic adaptation is affected in a controlled way. Another possibility to test our predictions would be to vary the firing rate of the neuron by increasing or decreasing the input current. In this way, the time scale of spiking would change relative to the time scale of adaptation and, thus, the colored noise effect of adaptation noise could be enhanced or attenuated, respectively.

Channel noise can crucially influence neural firing especially in the absence of synaptic input [58], [59]. This could be particularly relevant for the irregular discharge patterns of certain receptor cells. So far, channel noise has been studied mostly in the context of stochastic 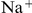 and 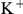 channel gating involved in the spike generation itself. These channels are considered to be fast. Because we were mainly interested in the effect of slow adaptation channels compared to fast fluctuations resulting from fast ion channels or synaptic activity, we lumped all fast noise sources into an unspecified additive white noise source. This is certainly a simplification; e.g. it has been shown in experiments that voltage noise due to 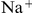 channels depends on the mean voltage itself [39], [40]. More detailed models of the various sources of noise are worth the efforts in future investigations. However, we do not expect that such sophisticated models would change our results qualitatively, because they mainly hinge upon the presence or absence of long time scales.

Realistic numbers of M-type channels per neuron are difficult to estimate and the numbers used in this paper must be seen as a tuning parameter for the channel noise intensity. Channel densities of the M-type have been estimated to be of the order of one functional channel per 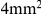 [60]. Assuming a spherical cell with a diameter of 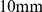 one obtains of the order of 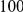 channels. Thus, the channel numbers used in this study (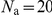
–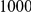) seem to be reasonable; and hence the M-current could be a potential source of fluctuations.

The diffusion approximation for the stochastic dynamics of ion channel populations (also known as Langevin or Gaussian approximation) has been studied by several authors [38], [61]–[64] (see also [65] in the context of chemically reacting particles). Here, we have shown how one can map the stochastic dynamics of a population of ion channels with negative feedback to the macroscopic current dynamics plus an additive colored noise (see [38] for a related treatment). In other words, the dynamics could be reduced to an analytically accessible Langevin equation for voltage and adaptation. In particular, we investigated the effect of the diffusion approximation on the statistics of interspike intervals and found a fairly good agreement with the channel model, despite the small number of channels. This seems surprising, given that for a typical parameter set – 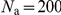, open probability 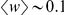 and 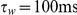 – one expects only 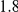 closing transitions (between spikes) and 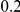 open transitions (during action potentials) per millisecond. Apparently, on the time scale of 1 ms the number of transition events is not Gaussian distributed. However, we found that the main effect of the channel noise consists in a slow modulation of the instantaneous firing rate on the large time scale of 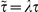, whereas high-frequency components of the noise are of minor importance. Thus, on relevant time scales of the order of 10–100 ms the average number of transitions is much larger and a Gaussian approximation seems to be reasonable.

Spike-frequency adaptation has been commonly studied with regard to its mean effect on the firing rate [18],[43]–[46]. It has been shown that these effects can be exhaustingly analyzed using a universal firing rate model [18]. In this paper, however, it became evident that higher-order statistics and fluctuation effects may differ and may be used to distinguish different kinds of noise sources.

## Methods

### Model for the stochastic adaptation current

**Figure 13:**
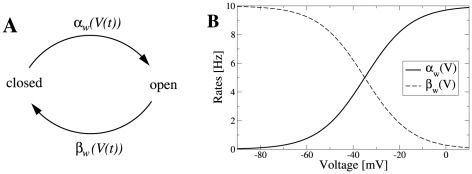
Channel model with voltage-dependent transition rates. **A** In a simple model of a M-type adaptation channel, the slow voltage-gated adaptation current is mediated by a population of slow ion channels that can be either in an open or a closed state. In the activation state (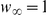), channels open with rate 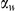, in the deactivation state (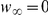) channels close with rate 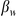. **B** Both transition rates show a sigmoidal dependence on the voltage (Eq. (24) and (27), 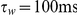).

To analyze the slow, voltage-dependent adaptation channels in a simple setup we consider a population of 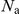 independent ion channels that reside in an open or a closed state. For each channel, we thus have the simple reaction kinetics shown in Fig. 13A.

For 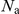 channels, one can either perform 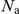 independent simulations of one two-state process or one simulation with 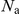 states where the number of open channels 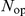 can be increased or decreased by one:
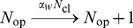
(20a)

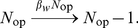
(20b)


Here, 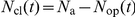 denotes the number of closed channels. The rates 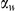 and 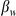 for the transitions between the closed and open state can be related to the (voltage-dependent) kinetics of a typical gating variable by choosing
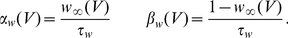
(21)


Therein, 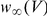 is the steady-state open probability of a single channel when the membrane potential is clamped at 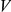 and 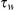 sets the time scale of the channel kinetics. Note, that both 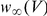 and 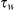 are accessible from experiments. The master equation for the open probability of the two-state model reads
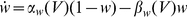
(22)or after insertion of Eq. (21)
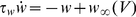
(23)which follows the common scheme for gating variables with voltage-dependent kinetics in Hodgkin-Huxley type models. We will use the function 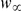 in two different versions.

In the conductance-based Traub-Miles model, we will use the kinetic scheme with the rate functions given by
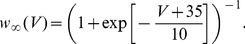
(24)


Inserting this relation into Eq. (21), both rate functions show a quasi sigmoidal dependence on the voltage (see Fig. 13B), such that 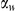 is appreciably different from zero only during the action potential (i.e. for 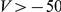
mV) whereas 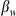 is only finite in the opposite range of a subthreshold membrane voltages.

For the integrate-and-fire (IF) model we employ a simplified variant that distinguishes only between two values of 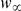: one attained when the voltage is subthreshold and another one for the duration of the action potential 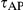. Specifically, at a spike event of the IF model, 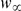 is set to one for a duration of 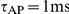 and otherwise it is set to zero (Fig. 1B). Thus, 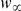 can be expressed as a function of time 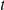 and the last spike time 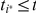:
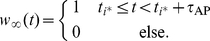
(25)


This function and the set of spike times 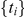 are related by
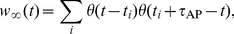
(26)where 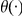 denotes the Heaviside function. Hence, in the simplified model, the dependence of the rates on the membrane voltage is substituted by an explicit dependence on time 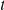 and the most recent spike time 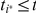:
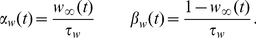
(27)


In the definition of 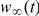, Eq. (25), we require that the duration of the pulse 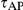 is much smaller than the mean ISI, so that overlaps of two subsequent pulses are unlikely. Note, that for simplicity channels can be activated only during spikes (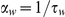), but not in between spikes (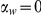). However, one can show that the following results are not changed qualitatively if one allows for subthreshold activation (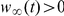), as observed for M-type potassium currents (see Discussion).

Taking the open probability exactly as the fraction of open channels amounts to taking the limit of an infinite population of channels. In the Traub-Miles model or the integrate-and-fire model with deterministic adaptation, this corresponds to adding an adaptation current of the form [18]

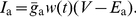
(28)


In this equation, 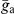 denotes the maximal conductance (per unit membrane area) and 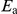 constitutes the reversal potential of the adaptation current.

For a finite channel population, the fraction of open channels is given by the stochastic quantity 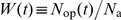, where 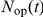 evolves according to the kinetic scheme Eq. (20). In contrast to Eq. (28), this gives rise to a stochastic adaptation current
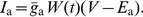
(29)


When the channel number is varied, we assume that the maximal conductance per unit membrane area 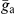 remains constant. Thus, a change of the channel number can be realized either by a variation of the total membrane area or by a change of the channel density in conjunction with a simultaneous scaling of the single channel conductance. Such procedures enable a change of the amount of channel noise, without changing the mean current per unit membrane area.

#### Diffusion approximation

The channel model, Eq. (20), can be approximated by a single Langevin equation for 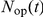 if the number of transitions in a sufficiently large time interval, which is still smaller than the decay time 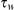, is treated as a Gaussian random variable [65]. Under this condition 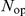 obeys
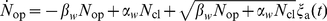
(30)with 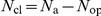 and Gaussian white noise 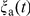, with 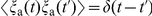. Dividing Eq. (30) by 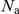 and using Eq. (27) we obtain a Langevin equation for the fraction of open adaptation channels 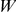,
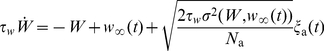
(31)where 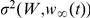 is given by
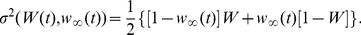
(32)


Furthermore, a separation of the adaptation into a deterministic and a stochastic part, 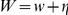, will be useful for the interpretation of our results. In these new variables Eq. (31) can be rewritten as two equations:
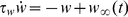
(33)

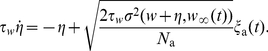
(34)


Note that Eq. (1), which will be our final diffusion model, also involves the additive-noise approximation presented below.

### Perfect integrate-and-fire model with adaptation

The perfect integrate-and-fire (PIF) model [2] constitutes a minimal model for a neuron possessing a stable limit cycle. In this model the subthreshold voltage is determined by the equation
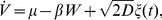
(35)where 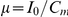 and 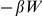 are proportional to the base current 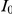 and adaptation current 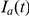, respectively; 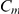 is the membrane capacitance and the scaling factor for the adaptation current reads 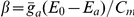. Here, we used an effective-time-constant approximation [66], where we substituted in Eq. (29) 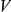 by the average voltage 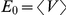 to obtain a voltage-independent adaptation current [18].

The last term in Eq. (35) represents fast Gaussian input fluctuations of intensity 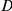 and correlation function 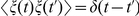 (here and in the following, the angular brackets denote an ensemble average). The model Eq. (35) is complemented by the fire-and-reset rule: upon reaching the threshold 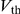 a spike is elicited and 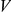 is reset to 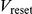, 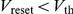. Because Eq. (35) is invariant with respect to a constant shift in 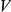, we can choose the reset value as the origin, i.e. 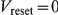. The threshold crossing events define the spike times 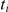, 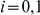,…of the PIF model.

It is a feature of the PIF model that the firing rate is directly proportional to a constant driving current and independent of the noise. However, even for the slowly varying driving current 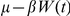 in Eq. (35), one can define an instantaneous firing rate
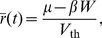
(36)which can be seen as the slowly varying firing rate that is obtained by averaging the spike train over the time scale 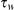 of the adaptation. Averaging over time scales much larger than 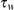 yields a constant firing rate 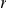, because the process is stationary. Thus, the (stationary) firing rate can be obtained by averaging Eq. (36), which gives 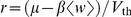. On the other hand, the firing rate is related to the mean fraction of open channels by averaging Eq. (33):
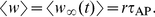
(37)


Solving for 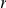 yields
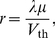
(38)where 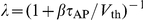.

#### Additive-noise approximation

The noise term in Eq. (31) or (34) is multiplicative, because both arguments of the noise strength 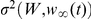 depend on the dynamical variables. For slow adaptation, 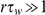, and large 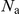, however, the relative fluctuations of 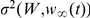 are small and, hence, 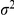 can be treated as constant (additive-noise approximation). This can be seen as follows: The rapid changes of 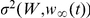 due to the switchings of 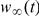 between 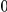 and 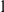 on the time scale of the mean ISI are averaged out by the linear filter dynamics Eq. (34) if 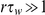. Thus, in Eq. (32) 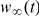 can be replaced by its local average 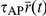. Because of Eq. (36), 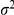 can therefore be written as a quadratic function of 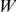. As explained below (Sec. “Colored noise approximation”), the variance of 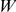 is 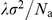. Thus, using 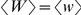 and 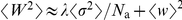 the mean of 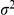 can be self-consistently determined. It can be shown that
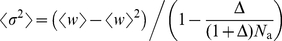
(39)

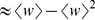
(40)

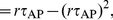
(41)where 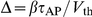 and 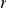 is given by Eq. (38). In the second line we neglected the term that depends on 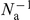, because for large 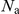 it is much smaller than unity. Similarly, the variance of 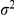 can be calculated neglecting terms that are third and forth order in 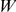. As a result, we obtain 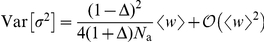. The relative fluctuations of the noise intensity are given by 
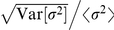
. Inserting the expressions for mean and variance of 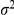 to first order in 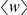, we find that the additive-noise approximation 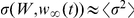 is roughly valid if
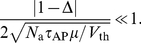
(42)


For the standard parameter set used in this paper, 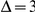, 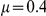, 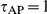, 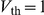, we find the condition 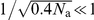. For instance, for 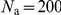 the relative fluctuations of 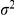 are 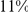.

#### Non-dimensional form

It is useful to regard a non-dimensional form of Eq. (35). To this end, we measure henceforth voltages in units of 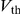 and time in units of 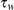, i.e. we introduce the variables 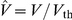 and 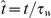. Furthermore, we will use the adaptation variable 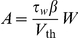. Then Eq. (31) and (35) can be rewritten as
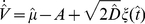
(43)

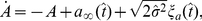
(44)where the firing threshold is 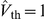 and the adaptation feedback is given by the function
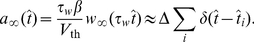
(45)


The dynamics of the PIF model is completely determined by the four non-dimensional parameters
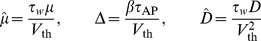
(46)and
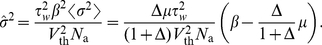
(47)


In the last step, Eqs. (38) and (41) were used. The second term of 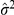 can be neglected, because it arises from the small term 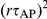 in Eq. (41). In fact, 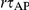 is the ratio between the spike duration and the mean ISI, which can be assumed to be small. Thus, the channel noise intensity is approximately
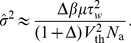
(48)


Again, the adaptation variable 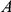 can be written as the sum of a deterministic part 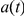 and a stochastic part 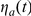, satisfying the equations
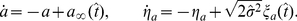
(49)


In accordance with Eq. (37), the mean adaptation is proportional to the firing rate [18],
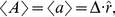
(50)where 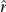 is the firing rate in units of 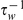.

#### Mean adaptation approximation

In the case of deterministic adaptation (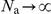), the membrane potential is driven by the current 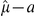 and by white noise. If the firing rate of the neuron is large compared to the decay rate of the adaptation current, i.e. if 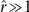, the trace 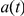 is effectively smoothed by the linear filter equation Eq. (49). As a consequence, the relative deviations of 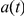 from its mean value 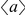 are small, so that the replacement 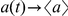 yields a good approximation for calculating the ISI density. In the PIF model, 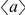 can be self-consistently determined from Eq. (50), because for the constant input current 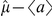 the firing rate of the PIF model is simply given by 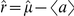. Solving for the firing rate yields
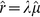
(51)with 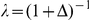. Thus, the mean adaptation approximation of the PIF model reads
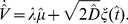
(52)


#### Colored noise approximation

In the case of stochastic adaptation (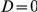) one can approximate the adaptation variable 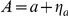 by an effective colored noise. If the adaptation is slow, i.e. 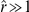, the spike train looks almost periodic at small time scales with a slowly varying frequency 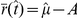 (in units of 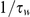), because of the absence of fast fluctuations. As a result, the instantaneous firing frequency 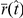 can be well estimated already by averaging over a few spikes. Because the linear filter in Eq. (49) averages 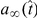 on time scales of the order of 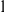, the dynamics of 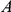 is retained if in Eq. (44) 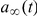 is replaced by its slowly varying average 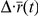:
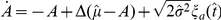



Multiplication with 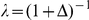 yields
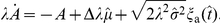



Finally, we perform the variable transformation 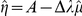, so that we obtain a colored noise driven PIF neuron model
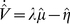
(53a)

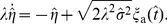
(53b)


As can be seen from Eq. (53a), the stationary firing rate for stochastic adaptation is again given by Eq. (51).

#### Nonstationary firing rate

If the stimulus depends on time, i.e. if 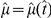, the firing rate is a function of time as well. Here, the firing rate 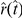 is understood in the sense of an ensemble average or peri-stimulus histogram. In the PIF model, such a firing rate is given by the ensemble average of the instantaneous rate 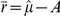, i.e. 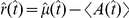. The ensemble average of 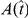 can be obtained by averaging Eq. (44). Solving for the firing rate yields
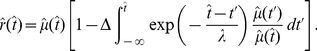
(54)


As an example, let us consider a step of the base current at time 
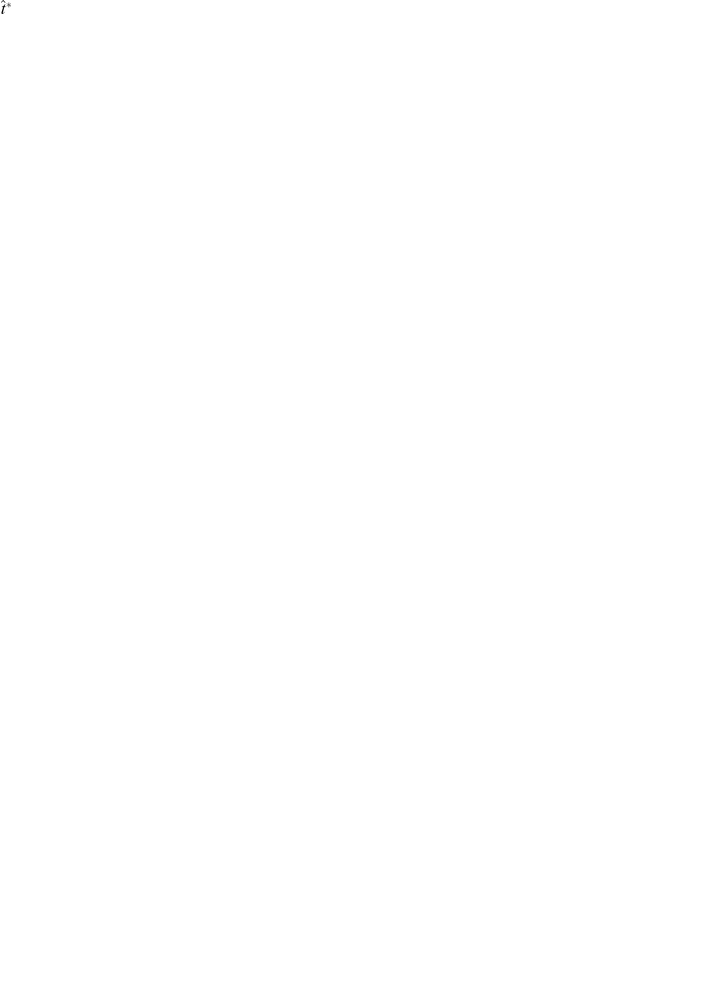
 such that 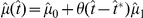. In this case, the time-dependent firing rate reads
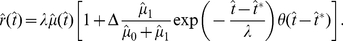
(55)


This represents exactly the exponential decay of the firing rate in response to a step stimulus, which is typical for spike-frequency adaptation. Eq. (55) has been used in Fig. 1C.

The dynamical response to a step stimulus yields an interesting interpretation of the scaling factor 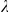: from Eq. (55) one finds that
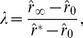
(56)which is the ratio of firing rate increases after adaptation to the increase directly after stimulus onset. In Eq. (56), 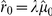, 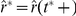 and 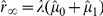 are the firing rates before stimulus onset, directly after the step and at 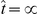, respectively. Thus, 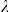 quantifies the “percentage adaptation” or the “degree of adaptation” [26].

### Statistical measures and known expressions for the PIF model

In the following, 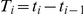 denotes the interspike intervals (ISIs), i.e. the intervals between adjacent spikes, and 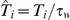 are the corresponding ISIs in units of the adaptation time constant. The statistics of ISIs can be characterized by different measures. A single interval 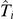 is distributed according to the probability density 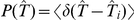, i.e. the normalized ISI histogram. The shape of the ISI density can be characterized using the cumulants. The first cumulant equals the mean ISI and the inverse of the firing rate 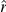,
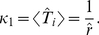
(57)


The second cumulant equals the variance and is related to the coefficient of variation (CV), which is a measure of ISI variability:
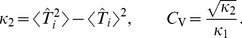
(58)


We further consider the third cumulant, which is related to the skewness 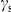 of the ISI distribution, defined as
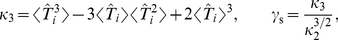
(59)and the fourth cumulant, which determines the kurtosis (or excess) 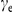 of the ISI distribution. It is defined as
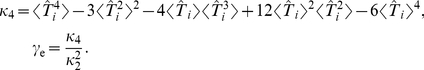
(60)


Roughly speaking, the kurtosis indicates how much of the variability is due to events that strongly deviate from the mean value. For instance, a unimodal ISI density with a heavier tail compared to another ISI density with the same CV, tends to exhibit a larger kurtosis. This is typically accompanied by a more pronounced peak close to the mean value to balance the heavy tail.

In this paper, we want to compare the ISI density with an inverse Gaussian probability density serving as a reference statistic. For an inverse Gaussian ISI distribution (see below) one observes that the skewness is proportional to the CV and the kurtosis scales like the squared CV. This suggests to introduce rescaled versions of the skewness and the kurtosis as follows:
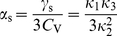
(61)and
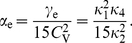
(62)


By defining the rescaled skewness and kurtosis in this manner, we obtain measures that are identical to unity for an inverse Gaussian ISI density. For larger (smaller) values, the ISI density is respectively more (less) skewed and more (less) peaked compared to an inverse Gaussian density. This procedure is somewhat analogous to the definition of the CV, for which the Poisson process serves as a reference for the ISI variability with 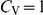.

Furthermore, we consider the correlations among ISIs as quantified by the serial correlation coefficient [67]:
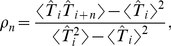
(63)where due to stationarity the expression does not depend on the integer 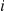, i.e. on the position in the sequence of ISIs. Alternatively, the averages involved in Eq. (63) can also be taken along the sequence (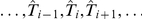).

For the adapting PIF model, the two limit cases that are considered in this paper could be reduced to simplified models, for which analytical results are partly known. Firstly, in the case of deterministic adaptation, i.e. 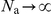, the ISI density can be approximately described by the first-passage-time density of a biased Brownian motion described by Eq. (52) (mean adaptation approximation). A classical result for this purely white noise driven PIF neuron with a constant drift 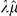 is that the ISI density is given by the so-called inverse Gaussian [2]

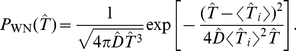
(64)


This distribution has a mean
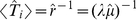
(65)and a CV
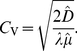
(66)


Furthermore, by construction we have
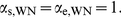
(67)


The mean adaptation approximation would wrongly predict that the ISIs are uncorrelated. The reason is that in the PIF model driven by only white Gaussian noise any memory of the ISI history is erased upon reset. A better account of ISI correlations is given below.

Secondly, the case of stochastic adaptation can be reduced to a PIF neuron driven by a reduced base current 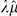 and a colored noise with correlation time 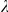 and variance 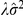 (Ornstein-Uhlenbeck process, see Eq. (53)). In the case of a weak colored noise, it is useful to define the small parameter
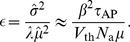
(68)


For 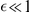 an approximation for the ISI density is given by [48]

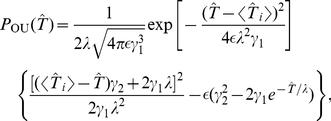
(69)


with 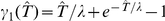, 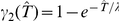 and mean ISI 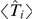. For 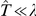, i.e. for ISIs much smaller than the correlation time of the colored noise, the expression for 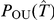 reduces to [50]:
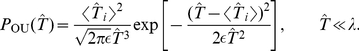
(70)


Although this formula captures the ISI density at small ISIs, it is of limited use, because the second and higher ISI moments diverge. Throughout the paper we have therefore used the full expression Eq. (69). The mean ISI and the firing rate do not depend on the noise statistics, in fact they are equal to the white noise case, Eq. (65), as shown below (derivation of the ISI cumulants). The squared CV can be obtained to second order in 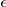 [48] using the methods presented below:
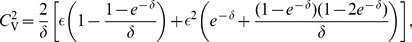
(71)with 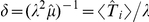. Similarly, the rescaled skewness and kurtosis are derived for weak colored noise below. Finally, the serial correlation coefficient can be computed analytically for weak noise [48] (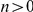):
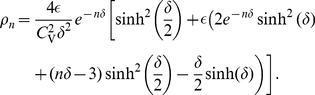
(72)


### Serial correlation coefficient of adaptive PIF neuron driven by white noise

Here we derive an expression for the serial correlation coefficient of a PIF neuron with deterministic adaptation current and white noise driving. We consider the following subthreshold dynamics
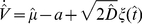
(73)

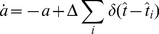
(74)augmented with the usual reset rule for 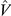 at the spike threshold 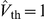. The adaptation variable 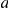 jumps by an amount 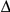 at each spiking event.

#### Deterministic limit cycle

**Figure 14:**
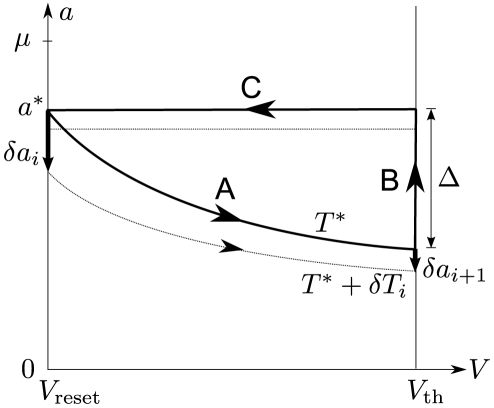
Illustration of the deterministic dynamics of the adaptive PIF neuron. The flow in the phase space spanned by 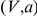 possesses a stable limit cycle, which consists of the voltage drift from 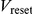 at 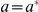 to the threshold (A), the following increase of 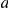 by an amount 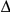 (B) and the subsequent voltage reset (C). The period of the limit cycle 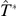 is solely due to the time of the process A, whereas the processes B and C occur instantaneously. A trajectory starting off the limit cycle at 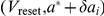 reaches the threshold at the time 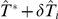. The deviation from 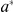 after one period has an absolute magnitude 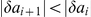.



If we set 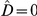 a trajectory in the plane spanned by the variables 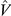 and 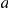 will converge to a stable limit cycle as sketched in Fig. 14. It consists of three segments: (i) the drift of 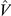 from reset to threshold which lasts for a time 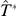 and the simultaneous decrease of 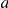 from 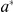 to 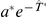, (ii) the instantaneous jump of 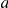 by an amount 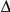 following the threshold crossing, and (iii) the instantaneous reset of 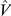. The time of the drift part 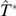 amounts to the whole period of the limit cycle, i.e. the interspike interval of the deterministic system. From this it is clear, that the limit cycle is determined by the two conditions

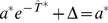
(75)and
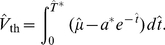
(76)


Solving for the limit cycle parameters 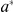 and 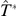 yields
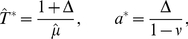
(77)where we introduced the abbreviation
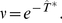
(78)


#### Deviations from the limit cycle due to a small perturbation

The serial correlation coefficient for the case of weak noise can be understood by considering small deviations from the limit cycle. Weak noise leads to a distribution of 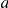 values upon reset, which is centered about 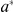. Hence, the value of 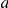 immediately after reset can be represented as 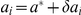, where 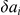 is a random number with 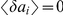. The index 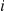 denotes the index in the sequence of interspike intervals, i.e. 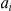 is the initial value of 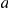 at the beginning of the 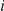
th ISI.

Similarly, noise leads to a distribution of ISIs centered about 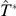 and for small noise intensities the mean ISI coincides with 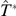. Thus, the 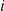
th ISI can be represented as 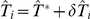, where 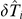 is a random number with 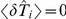. The deviation from the limit cycle after one period, 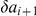, is a function of 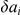 and 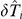. A simple calculation reveals that
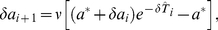
(79)which after expanding 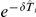 to first order in 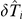 and omitting the higher-order term 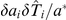 reduces to
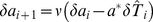
(80)or solving for 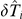:
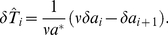
(81)


The last equation allows to express the interval correlations 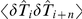 in terms of the correlations 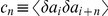:
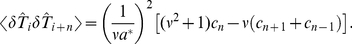
(82)


Note, that 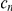 is symmetric, in particular, 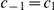.

#### Correlations of subsequent deviations from the limit cycle

In order to calculate the correlations 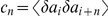 the statistics of 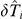 for a given initial perturbation 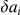 is needed. To this end, we assume that 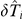 can be represented in the following form:
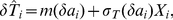
(83)where 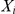 is an independent random number with 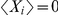 and 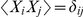. The functions 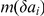 and 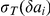 denote for a fixed 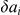 the mean deviation of 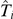 from 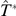 and the standard deviation of 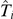, respectively. The representation Eq. (83) is indeed justified, because in the weak-noise limit the distribution of 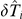 for fixed 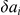 is close to a Gaussian distribution. Since any Gaussian random variable is completely determined by its first two moments, it can always be put in the form Eq. (83). Furthermore, for small perturbations 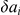 one can expand the mean deviation up to first order yielding 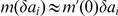 (the zeroth-order term must vanish, since 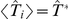 for 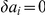).

Inserting Eq. (83) into Eq.(80) yields a stochastic map for 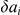:
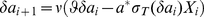
(84)where we defined
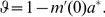
(85)


Multiplying Eq. (84) by 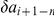 and averaging over the full ensemble gives
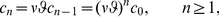
(86)where we have taken into account that for 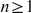 the perturbation 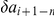 is uncorrelated with 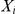.

**Mean ISI for a given perturbation** 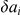. It remains to determine 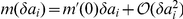, i.e. how much the mean ISI is changed by a small perturbation 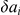. To this end, we integrate Eq. (73) from reset to threshold and obtain
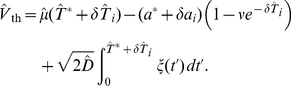
(87)


Linearizing the exponential 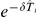 and averaging Eq. (87) over an ensemble with fixed 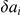 (neglecting the higher-order noise term) yields
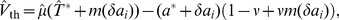
(88)which after subtraction of the unperturbed equation (Eq. (76)) leads to
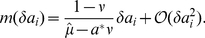
(89)


We mention, that this expression is consistent with the result in [68] in the limit of vanishing noise and 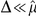. Inserting the linear coefficient 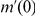 into Eq. (85) gives therefore an expression for 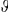:
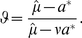
(90)


Using Eq. (82) and Eq. (86) in the expression for the serial correlation coefficient 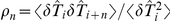, 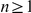, leads to Eq. (9). Our result is the limiting expression for vanishing noise (in fact, Eq. (9) does not depend on the noise intensity at all). Corrections for moderate noise intensity may be feasible with the results in [68].

### ISI cumulants of a PIF neuron driven by colored noise

Following [48], we derive here the ISI cumulants of the colored-noise driven PIF model, Eq. (53), in the weak-noise approximation. The ISI cumulants are required to compute the rescaled skewness and kurtosis. The Fokker-Planck equation associated to Eq. (53) for the time-dependent joint probability density 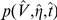 reads
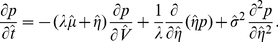
(91)


For the description of the ISI density it is necessary to use the initial condition that corresponds to the distribution of 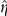 upon spiking. The initial condition is for weak noise, 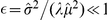, well approximated by (see [48])
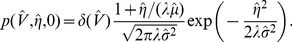
(92)


The ISI density is equal to the time-dependent probability flux across the threshold line 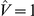 if threshold crossings of trajectories with negative 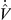, i.e. crossing from above the threshold, are prohibited. This is achieved by imposing a reflecting boundary on the half line 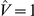, 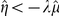. For 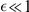, however, negative 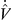 are highly unlikely, so that the free process without reflecting boundary generates a flux that is a reasonable approximation of the ISI density.

To carry out a weak-noise expansion of Eq. (91) we change to the variables
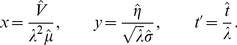
(93)


In these variables the Fokker-Planck equation for 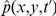 reads
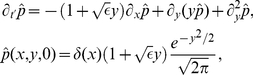
(94)where 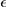 is given by Eq. (68). The density for the ISIs (in units of 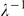) is approximately the total probability flux across the threshold:
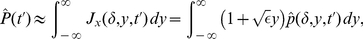
(95)where
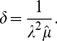
(96)


Furthermore, we consider the characteristic function 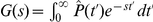, which by means of Eq. (95) can be expressed as
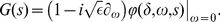
(97)


In the last equation we introduced the function
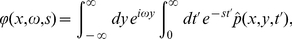
(98)which arises from the subsequent application of a Laplace and a Fourier transformation to the probability density 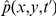. The cumulants 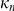 of the ISI density can be obtained from the characteristic function, see e.g. [69]:
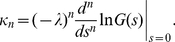
(99)


From Eq. (97) and (99) it is clear, that all cumulants can be generated from the function 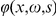. Applying the Laplace and Fourier transformation (as in Eq. (98)) to Eq. (94) we arrive at an equation for 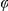, which reads
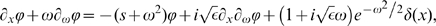
(100)with the boundary condition
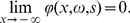
(101)


To solve Eq. (100) for weak noise, we expand 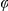 in powers of the small parameter 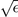:
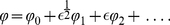
(102)


Inserting into Eq. (100) gives a hierarchy of first-order differential equations for the coefficients 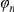:
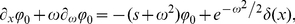
(103)

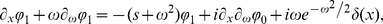
(104)

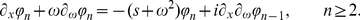
(105)


The solutions can be obtained order-by-order using the method of characteristics. Here, we report only the first three coefficients 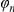:
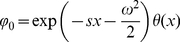
(106)

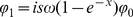
(107)

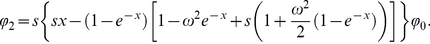
(108)


Higher-order coefficients can be calculated in the same way, although the expressions become quite lengthy. To obtain the kurtosis including the first noise-dependent term correctly, one has to calculate 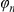 up to the eighth order. These straightforward computations can be accomplished by using a computer algebra system. As a result, the cumulants up to fourth order in 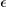 read
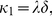
(109)

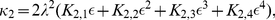
(110)

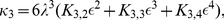
(111)

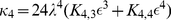
(112)with
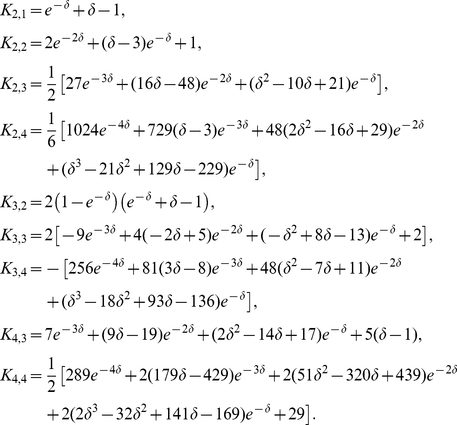



From the cumulants one finds the rescaled skewness and kurtosis as defined in Eq. (61) and (62):
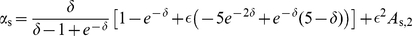
(113)and
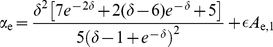
(114)with
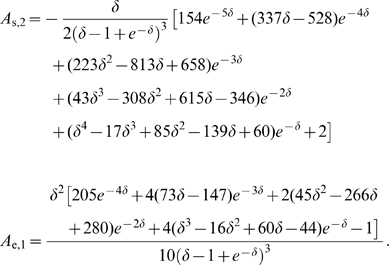



### Traub-Miles model with adaptation

To test the generality of our findings, we simulated the conductance-based Traub 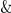 Miles model modified by B. Ermentrout [51]. It is a single compartment model with an additional M-type current, i.e. a slow voltage-dependent potassium current, inducing spike-frequency adaptation. In order to contrast the effects of deterministic versus stochastic adaptation on the firing statistics of the conductance-based model, we simulated two versions with either additive white Gaussian noise or adaptation channel noise. For the first model with fast fluctuating current noise and deterministic adaptation, the membrane potential 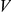 measured in mV is determined by
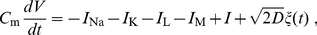
(115)where 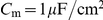 denotes the membrane capacitance, 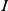 is the base current, and 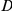 indicates the intensity of Gaussian white noise with correlation function 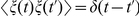. The deterministic ionic currents are given by the following equations [51]:

**Sodium current:** 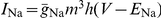



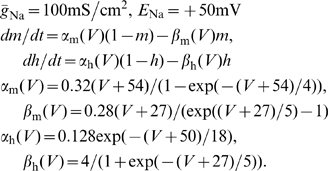
 **Potassium current:** 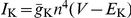


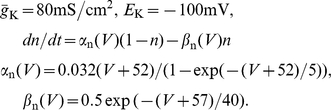



**Leak current:** 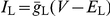


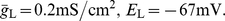



**M-type adaptation current:** 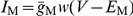


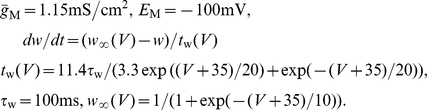



In this model, the adaptation time constant 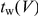 is a voltage-dependent function that we reparameterized such that 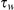 roughly corresponds to the time constant governing the exponential buildup of 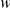 during periodic firing at 100 Hz in a simulation of equation (123) without the 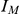 current and with 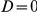.

For the second model with adaptation channel noise, the voltage is described by
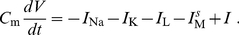
(116)


The currents 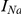, 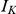 and 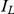 are the same as in the first model. The M-type adaptation current, however, is modeled as a stochastic current 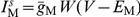 where 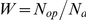 indicates the fraction of open channels.

As in the PIF model, we assumed the adaptation channels to be two-state ion channels with the transition rates 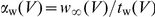 and 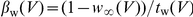. The gating of the adaptation channels was simulated using the Gillespie algorithm [70], [71]. This algorithm calculates the time interval until the next state transition, determines the reaction type, here channel opening or closing, and updates the number of channels in each possible state accordingly. For a given time step, the number of channels 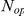 in the open state is then used to calculate the fraction of open channels 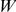 as well as the stochastic adaptation current 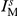. Furthermore, since the transition rates depend on 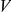, we restricted the maximal transition time to 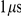.

In the model with stochastic adaptation current, the maximal channel conductance 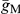 and the constant base current (
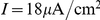
) were chosen to result in a CV 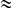 0.6 and a firing rate of 100 Hz for a simulation of 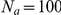 ion channels carrying the adaptation current. For the simulation with deterministic adaptation current and additive white noise the base current was adjusted (see corresponding figure captions) to yield the same rate while keeping the conductance 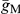 the same.
